# Neutrophil pyroptosis regulates corneal wound healing and post‐injury neovascularisation

**DOI:** 10.1002/ctm2.1762

**Published:** 2024-11-04

**Authors:** Peng Chen, Zhentao Zhang, Lilian Sakai, Yanping Xu, Shanzhi Wang, Kyung Eun Lee, Bingchuan Geng, Jongsoo Kim, Bao Zhao, Qiang Wang, Haitao Wen, Heather L. Chandler, Hua Zhu

**Affiliations:** ^1^ Department of Surgery The Ohio State University Wexner Medical Center Columbus Ohio USA; ^2^ College of Pharmacy and Health Sciences St. John's University Queens New York USA; ^3^ Department of Microbial Infection and Immunity The Ohio State University Columbus Ohio USA; ^4^ College of Optometry The Ohio State University Columbus Ohio USA

**Keywords:** corneal wound healing, neovascularisation, neutrophil, pyroptosis

## Abstract

**Rationale:**

The cornea is a unique structure that maintains its clarity by remaining avascular. Corneal injuries can lead to neovascularisation (CNV) and fibrosis and are the third most common cause of blindness worldwide.

**Objective:**

Corneal injuries induce an immune cell infiltration to initiate reparative processes. However, inflammation caused by sustained immune cell infiltration is known to be detrimental and can delay the healing process. This study was designed to understand the potential role of neutrophil and epithelial cell crosstalk in post‐injury CNV.

**Methods and results:**

Western blotting and immunostaining assays demonstrated that neutrophils infiltrated corneas and underwent pyroptosis following acute alkali injury. In vivo studies showed that genetic ablation of Gasdermin D (GsdmD), a key effector of pyroptosis, enhanced corneal re‐epithelialisation and suppressed post‐injury CNV. In vitro co‐culture experiments revealed that interleukin‐1β (IL‐1β) was released from pyroptotic neutrophils which suppressed migration of murine corneal epithelial cells. Real‐time RT‐PCR and immunostaining assays identified two factors, Wnt5a and soluble fms‐like tyrosine kinase‐1 (sflt‐1), highly expressed in newly healed epithelial cells. sflt‐1 is known to promote corneal avascularity. Bone marrow transplantation, antibody mediated neutrophil depletion, and pharmacological inhibition of pyroptosis promoted corneal wound healing and inhibited CNV in an in vivo murine corneal injury model.

**Conclusion:**

Taken together, our study reveals the importance of neutrophil/epithelium crosstalk and neutrophil pyroptosis in response to corneal injuries. Inhibition of neutrophil pyroptosis may serve as a potential treatment to promote corneal healing without CNV.

**Key points:**

- Neutrophil pyroptosis delays re‐epithelialization after corneal injury
- Compromised re‐epithelialization promotes corneal neovascularization after injury
- Inhibition of post‐injury pyroptosis could be an effective therapy to promote corneal wound healing.

## Graphical abstract

• Neutrophil pyroptosis delays re‐epithelialisation after corneal injury;

• Compromised re‐epithelialisation promotes corneal neovascularisation after injury;

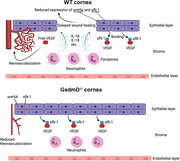

## 1 INTRODUCTION

Ocular injuries account for 3%−4% of work related injuries and 7%−18% of ocular traumas in the United States, and chemical burns comprise the majority of these injuries.^1^ Each year, about 36 000 chemical burns have been reported from emergency departments,^2^ and while accidents leading to ocular burns occur at all ages, individuals between 18 and 64 years of age are most commonly affected.^3^

Chemical burns to the eye are usually caused by either alkaline or acidic agents.^4^ Although both are serious injuries, alkaline burns are more common^5^ and cause more severe damage than acid burns.^6^ The systemic response to tissue damage and the subsequent wound healing response typically results in induction of inflammatory cascades. Neutrophils provide the first response to tissue damage and may have dual functions. Initially, the neutrophils infiltrate into injured tissue and protect wounds from invading pathogens and clears debris.^7^ However, due to the short half‐life of infiltrating neutrophils, dead neutrophils can release their nuclear and granular contents, known as neutrophil extracellular traps (NETs). Release of NETs can inhibit keratinocyte migration, possibly proliferation,^8^ impair the wound healing process, and can promote corneal neovascularisation (CNV).^9^, ^10^, ^11^ Thus, understanding the molecular mechanisms associated with neutrophil death is critical for the development of potential treatments that enhance tissue repair and suppress the potential detrimental actions of neutrophils following corneal injury.

Neutrophils have been shown to release IL‐1β following pyroptosis, particularly in inflamed tissues or under conditions of cellular stress.^12^, ^13^ Caspase‐1 plays a major role in the activation of the pro‐inflammatory cytokines IL‐1β and IL‐18 in the neutrophil.^14^ Caspase‐1/11 knockout and IL‐1β and IL‐18 knockout can improve the survival rate in both the cecal ligation and puncture and septic shock mouse models.^15^, ^16^, ^17^ Within the cornea, IL‐1β has been demonstrated to promote CNV and when IL‐1β is neutralised, CNV is inhibited.^18^ Further, multiple ocular surface disease models demonstrate increased IL‐1β and IL‐18, in which both pro‐inflammatory cytokines are associated with decreased corneal epithelial viability.^19^, ^20^, ^21^, ^22^, ^23^, ^24^ These studies highlight the important role of inflammatory cytokines released in disease states.

Pyroptosis, a lytic form of cell death, is a key pathway triggering inflammation.^25^, ^26^, ^27^, ^28^ Gasdermin D (GsdmD) has been confirmed as the key effector leading to pyroptosis and NETosis.^29^, ^30^ Neutrophil NETosis requires GsdmD formed pores for the rupture of the plasma membrane and granule, with subsequent NET extrusion.^26^, ^31^ In addition to activation of IL‐1β and IL‐18, caspase‐1 is required and involved in the cleavage of GsdmD.^29^, ^32^, ^33^ Recently, cleavage of GsdmD and induction of neutrophil pyroptosis via caspase‐11 and neutrophil elastase has also been reported.^34^, ^35^, ^36^ Others have shown that alkali injury to the cornea upregulates GsdmD, with concurrent increases in IL‐1β, IL‐18, and caspase‐1; inhibition of pyroptotic signalling resulted in improved healing.^20^, ^37^ However, to our knowledge, the involvement of neutrophil pyroptosis in corneal wound healing is largely unknown.

Here, we revealed that genetic ablation of GsdmD (*GsdmD^−/−^*) could promote corneal wound healing and reduce subsequent CNV. Mechanistically, IL‐1β released from pyroptotic neutrophils suppressed migration of corneal epithelial cells and delayed corneal healing. Furthermore, we identified two important molecules, Wnt5a and sflt‐1, both of which were highly expressed in the newly differentiated epithelial cell after injury. Thus, inhibiting neutrophil pyroptosis and promoting sflt‐1 expression facilitates timely corneal reepithelisation and suppression of post‐injury CNV. Our study highlights the importance of neutrophil pyroptosis in corneal wound healing and neovascularisation; pyroptosis may be a potential target for developing effective means to treat corneal wounds.

## 2 RESULTS

### 2.1 GsdmD deficiency promotes corneal wound healing and reduces neovascularisation

**FIGURE 1:**
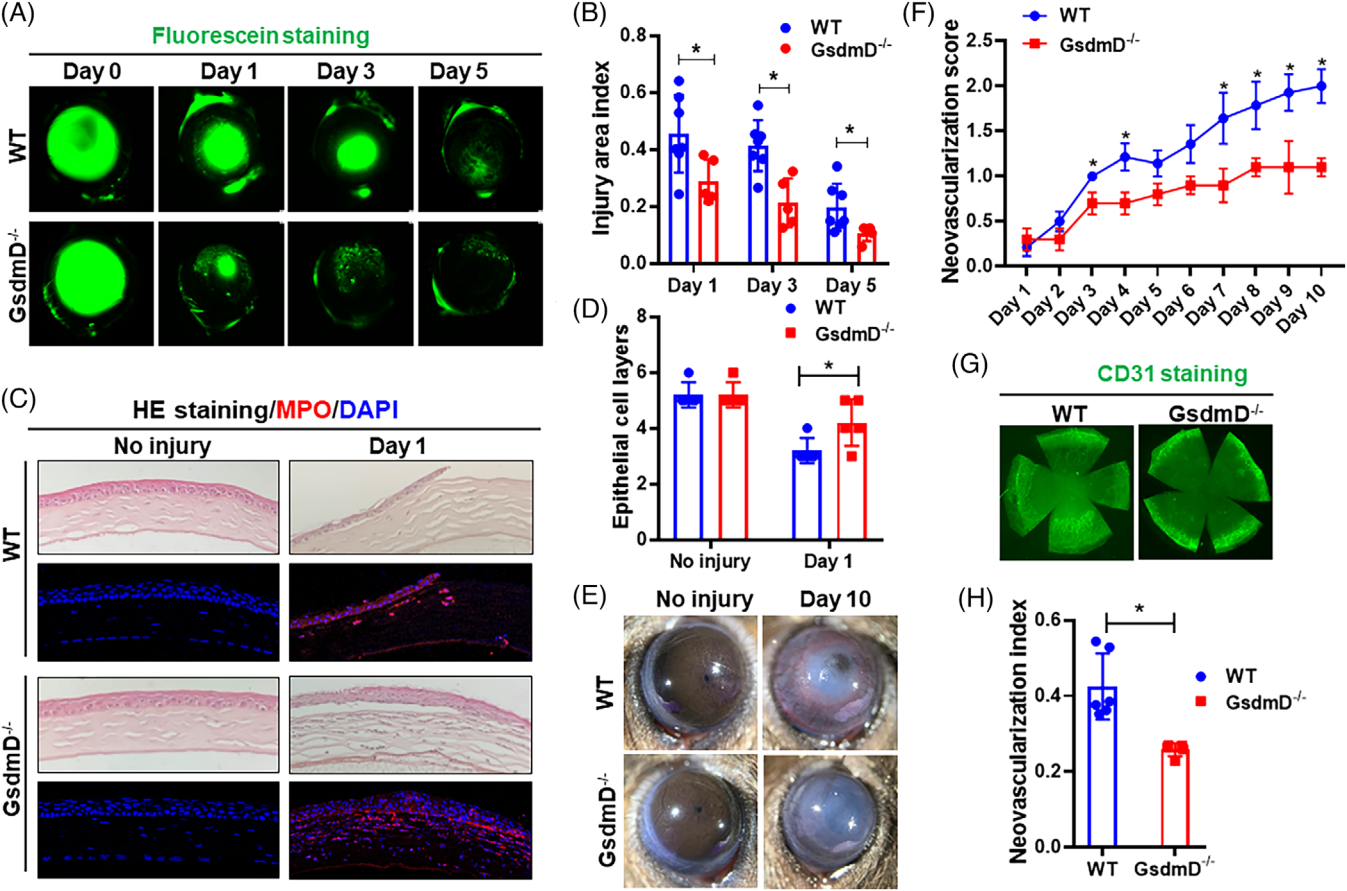
***GsdmD^−^^/^^−^*** corneas heal faster and develop less neovascularisation after alkali induced injury. (A) Representative images of fluorescein uptake show that re‐epithelialisation was improved in the corneas of *GsdmD* ***^−^^/^^−^*** mice as compared to WT mice (*n* = 5 for *GsdmD* ***^−^^/^^−^*** mice, *n* = 7 for WT mice). (B) Quantification of fluorescein signal. (C) Representative images of H&E and immunofluorescence staining show that *GsdmD* ***^−^^/^^−^*** corneas have improved re‐epithelialisation with significantly more epithelial cell layers one day after injury. MPO staining indicating that more neutrophils infiltrated the *GsdmD^−/−^* mice, as compared to WT corneas. (D) Quantification of corneal epithelial cell layers. (E) At day 10 post alkali injury, *GsdmD^−/−^* corneas developed significantly less CNV, as compared to WT corneas. (F) Neovascularisation scores at indicated time points. (G) CD31 staining of flat mount corneas shows that corneas derived from *GsdmD* ***^−^^/^^−^*** mice had less vascularisation than those from WT mice. (H) Quantification of CD31 fluorescent area. Data represent one experiment representative of two independent experiments (A–H). WT, Wild type; MPO, myeloperoxidase; CNV, corneal neovascularisation; H&E, Hematoxylin–Eosin.

To study the potential role of pyroptosis in corneal wound healing, we used GsdmD deficient (*GsdmD^−/−^*) mice and subjected them to alkali induced corneal injury.^38^ Twenty‐four hours after injury, corneal fluorescein staining demonstrated that *GsdmD^−/−^* mice (28.89% ± 7.63%) had significantly smaller wound areas than that of wild‐type (WT) mice (45.57% ± 13.60%) (Figure 1A,B). Consistent with the fluorescein staining, Hematoxylin–Eosin (H&E) and 4',6‐diamidino‐2‐phenylindole (DAPI) immunofluorescent (IF) staining also showed improved corneal re‐epithelisation in the *GsdmD^−/−^* mice (Figure 1C,D). The number of epithelial cell layers in the GsdmD KO mice (4.2 ± .8) was significantly higher than that of WT mice (3.2 ± .4). The myeloperoxidase (MPO) levels were similar between the two groups (Figure 1C).

Alkali injury is a well‐established model for the induction of CNV.^39^ After injury, limbal blood vessels are stimulated to grow centrally towards the axial cornea, compromising vision. Following injury induction, healing and vascular encroachment were examined daily until day 10. Representative images of mouse eyes clearly show that CNV was significantly reduced in *GsdmD^−/−^* corneas compared to WT corneas (Figure 1E,F). CD31 staining of flat mount corneas further confirmed a reduction of CNV in injured *GsdmD^−/−^* corneas (Figure 1G,H). Interestingly, we failed to observe significant differences in fibrosis between WT and *GsdmD^−/−^* mice (Figure S1). Thus, our findings suggest that GsdmD plays a detrimental role in corneal re‐epithelialisation and vascularisation but may not be involved in mitigating stromal fibrosis.

### 2.2 Neutrophils are the main cell type that undergo pyroptosis after corneal wounding

**FIGURE 2:**
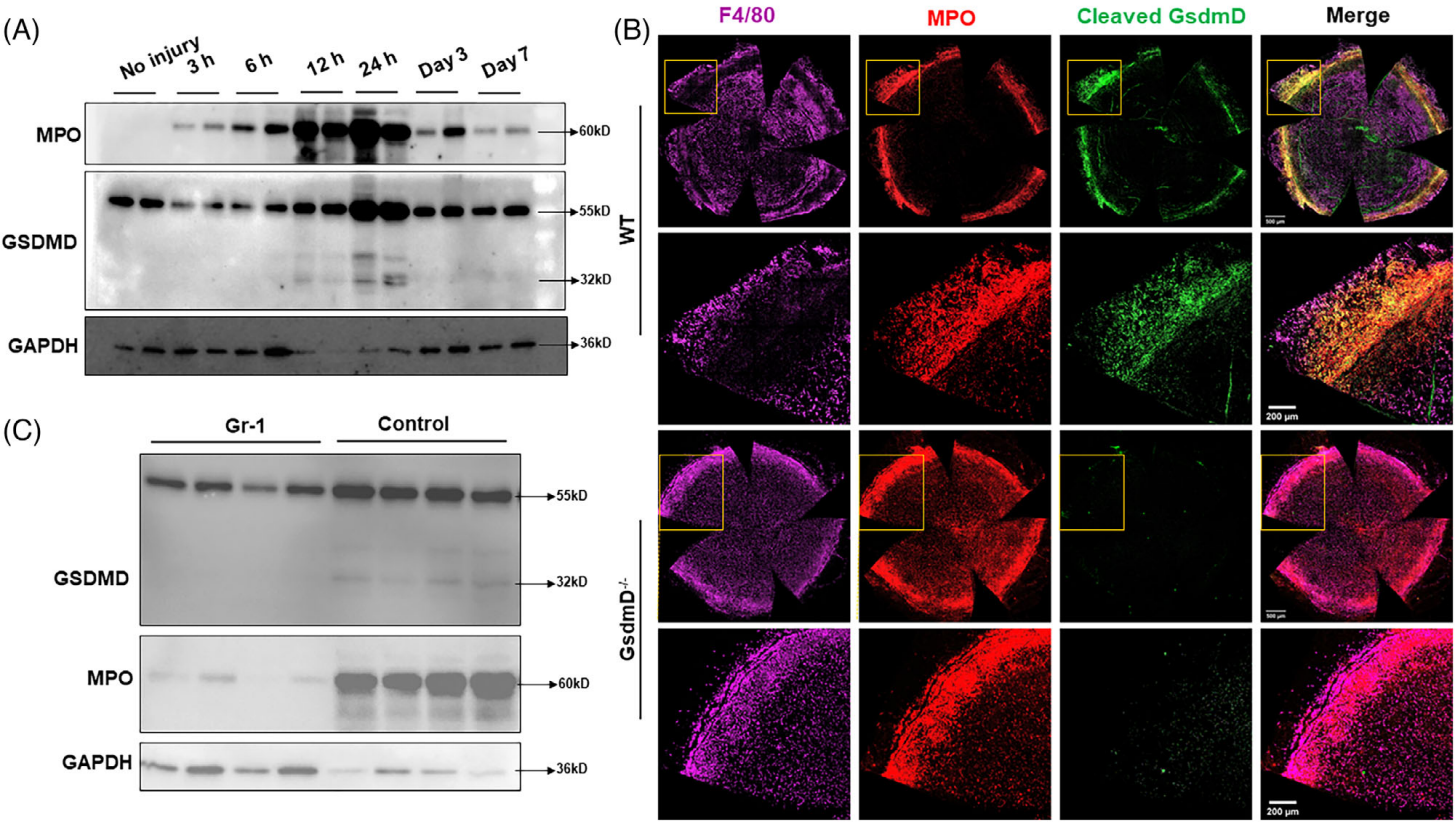
Neutrophils infiltrate into injured corneas and undergo pyroptosis. (A) Western blotting analysis showed expression of MPO and GsdmD protein in the injured corneas of WT mice at indicated time points after alkali injury. The number of mice used at each time point ranges from 5 to 7. (B) Representative immunofluorescent images of flat mount corneas were stained with cleaved GsdmD (GsdmD‐N), F4/80, and MPO. Co‐localisation of MPO and GsdmD‐N was detected. The corneas were stained 24 h after injury. (C) WT mice receiving Gr‐1 antibody treatments had a dramatic reduction in MPO and GsdmD‐N expression at 24 h after alkali induced injury (*n* = 4 for each group). Data represent one experiment representative of two independent experiments (A–C). WT, Wild type; MPO, myeloperoxidase.

As the corneal epithelium is the initial cell type affected by alkali injury, we first tested whether corneal epithelial cells underwent pyroptosis. However, we failed to induce pyroptosis in primary cultured mouse corneal epithelial cells (mCEC) with a combination of lipopolysaccharide (LPS) and nigericin (Ni) treatment (Figure S2). To further delineate the source of pyroptosis after corneal wounding, we next determined the dynamics of pyroptosis after corneal injury. As shown in Figure 2A, activation of pyroptosis (cleaved GsdmD: GsdmD‐N) was observed as early as 12 h after injury and expression peaked at 24 h. Interestingly, when we probed the same samples with MPO (a neutrophil marker), we observed neutrophils as early as 3 h after injury and peaked at the same time (24 h) as pyroptosis (Figure 2A), indicating a possible link between neutrophil infiltration and pyroptosis. Co‐staining for F4/80 (a macrophage marker), MPO, and GsdmD‐N demonstrated that activation of pyroptosis predominantly co‐localised with neutrophils, and less so with macrophages (Figure 2B). Additionally, we observed that neutrophils were localised mainly around the limbus in WT mice at 24 h after injury, while a greater number and wider distribution (both in the limbus and cornea) of neutrophils was detected in *GsdmD^−/−^* eyes (Figure 2B). To determine the extent that neutrophils contributed to post‐injury pyroptosis in the cornea, we then injected the mice with Gr‐1 antibody to deplete neutrophils prior to injury.^40^ As shown in Figure 2C, depletion of neutrophils almost completely abolished activation of pyroptosis, as evidenced by lack of GsdmD‐N signal. Taken together, these results indicate that alkali injury‐induced pyroptosis largely occurred in infiltrating neutrophils.

To further confirm neutrophils undergo pyroptosis, we isolated and purified primary neutrophils from mouse bone marrow. We found that isolated neutrophils underwent pyroptosis quickly after isolation followed by activation of apoptosis, as evidenced by cleaved caspase‐3 (Figure S3). We also found that treatment with disulfiram (a reported pyroptosis inhibitor) could significantly inhibit GsdmD cleavage, but not Caspase‐3 cleavage, in isolated neutrophils (Figure S4). Thus, these findings demonstrate that pyroptosis is an important type of cell death in neutrophils.

### 2.3 Neutrophil pyroptosis delays epithelial cell migration in vitro

**FIGURE 3:**
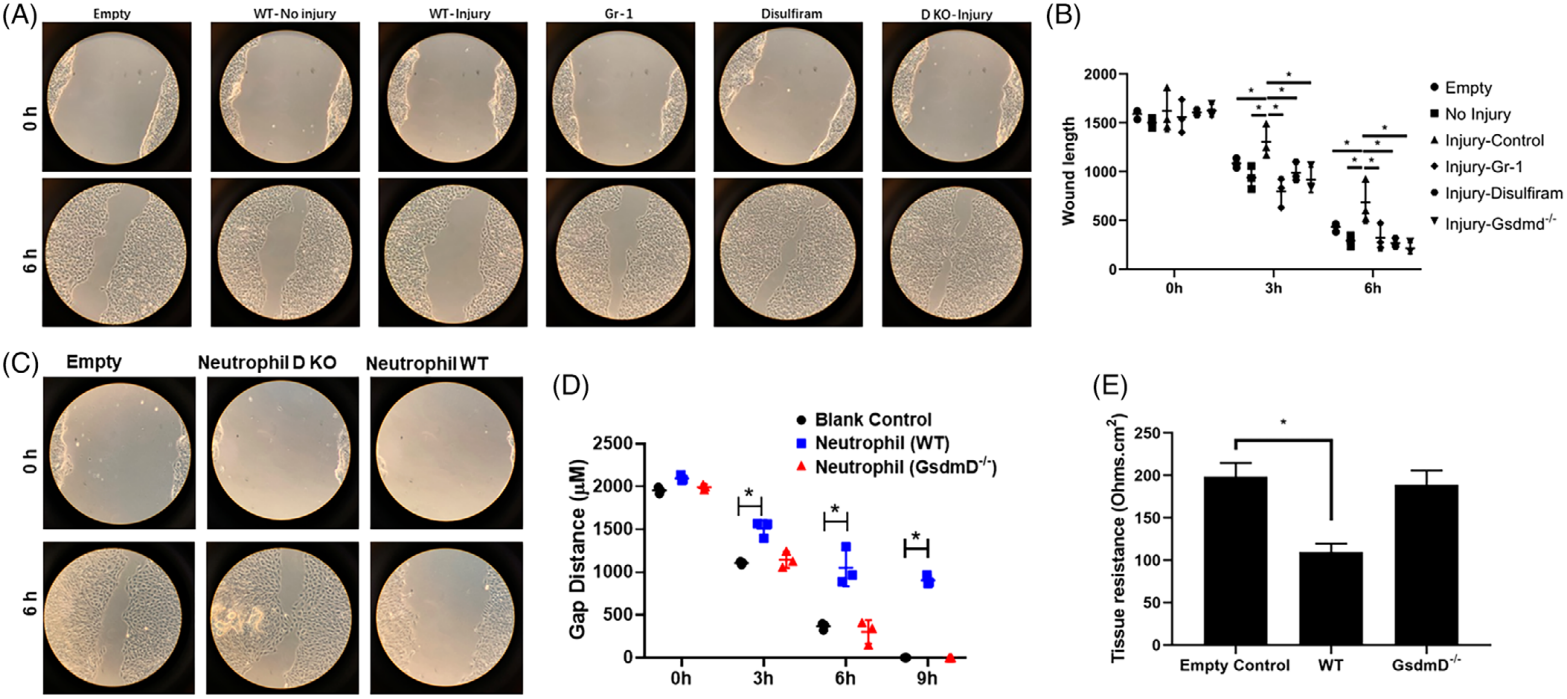
Pyroptotic neutrophils inhibit corneal epithelial cell migration and reduce monolayer integrity. (A) Corneas from indicated treatments were incubated with corneal epithelial cells. Scratch wounds were induced and representative images were taken at 0 and 6 h after scratch wound. (B) Quantification of scratch wounds. (C) Co‐culture of isolated neutrophils derived from WT mice, but not *Gsdmd−/−* mice, reduced migration of corneal epithelial cells; (D) quantification of migration rates. (E) Co‐culture of WT neutrophils with corneal epithelial cells disrupted epithelial integrity, as measured by TEER. Data represent one experiment representative of three independent experiments (A–E). WT, wild type; TEER, transepithelial electrical resistance.

We next tested the potential function of neutrophil pyroptosis in corneal wound healing. We first dissected mouse corneas, with or without injury, from WT, *GsdmD^−/−^*, Gr‐1 treated, and disulfiram treated mice, and cultured the dissected corneas with mCECs. A scratch wound was then created in the mCEC. As shown in Figure 3A,B, injured WT corneas significantly delayed wound closure of mCECs, as compared to the cells incubated with non‐injured WT control corneas. Furthermore, when we used injured corneas from Gr‐1 treated (Gr‐1 in Figure 3A), disulfiram treated (disulfiram in Figure 3A), and *GsdmD^−/−^* (D KO‐injury in Figure 3A) mice, the inhibitory effects on mCECs migration were greatly reduced (Figure 3A,B). These results suggest that injured corneas can suppress mCEC migration.

To test whether these factors were derived from neutrophils, we co‐cultured mCECs with isolated primary mouse neutrophils from either WT or *GsdmD^−/−^* mice. As shown in Figure 3C,D neutrophils derived from WT mice significantly delayed wound closure of mCECs, as compared to *GsdmD^−/−^* neutrophils. Furthermore, transepithelial electrical resistance (TEER) measurements indicate that co‐culturing mCECs with WT neutrophils can impair the structural integrity of epithelial junctions, as compared to *GsdmD^−/−^* neutrophils (Figure 3E).

As previously reported, IL‐1β and IL‐18 are two main cytokines released by immune cells after pyroptosis.^41^, ^42^ We hypothesised that IL‐1β release from pyroptotic neutrophils would be associated with suppressed epithelial cell migration. Indeed, IF staining showed increased IL‐1β signal after injury and this signal mainly co‐localised with MPO in the cornea and limbus (Figure S5).

**FIGURE 4:**
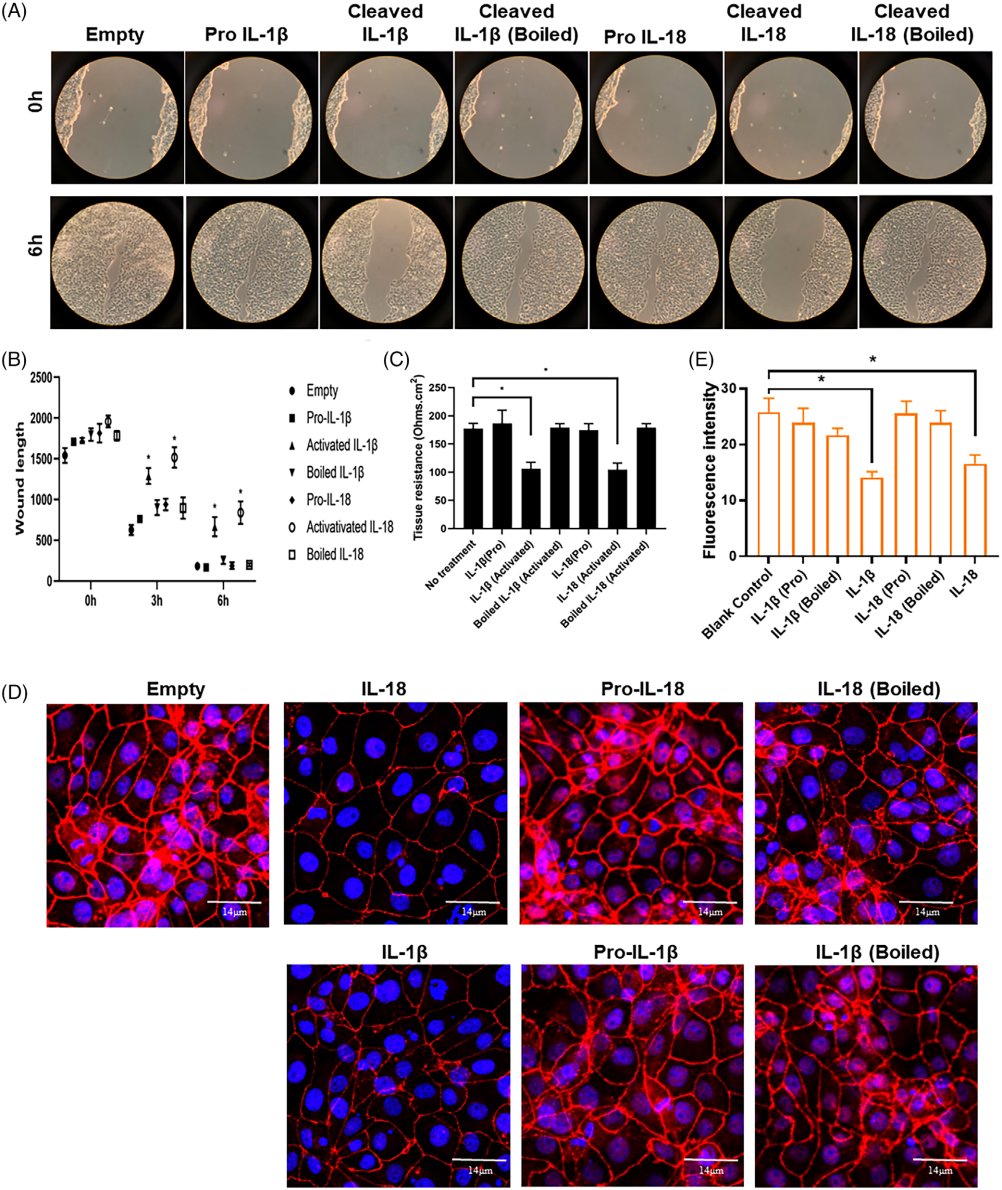
IL‐1β and IL‐18 inhibits corneal epithelial cell migration and reduces monolayer integrity. (A) Treatment of recombinant activated IL‐1β and IL‐18 proteins significantly reduced corneal epithelial cell migration. Pro and boiled recombinant IL‐1β and IL‐18 proteins were used as controls. (B) Quantification of migration rates. (C) TEER measurements showed that activated IL‐1β and IL‐18 could significantly reduce the electrical resistance of corneal epithelial cells. (D) ZO‐1 staining demonstrates disruption of tight junctions after treatment with activated IL‐1β and IL‐18 recombinant proteins. (E) Quantification of ZO‐1 fluorescent intensity in each group. Data represent one experiment representative of three independent experiments (A–E). IL, Interleukin; TEER, transepithelial electrical resistance.

We then generated recombinant pro‐IL‐1β and pro‐IL‐18 proteins from *E. coli*. The proteins were treated with caspase 1 to obtain the active forms. We found that treatments of active IL‐1β and IL‐18 significantly delayed mCEC wound closure, while treatments of pro‐ or boiled IL‐1β and IL‐18 failed to alter mCEC migration (Figure 4A,B). Similarly, active IL‐1β and IL‐18 disrupted epithelial cell tight junctions, as measured by TEER (Figure 4C) and ZO‐1 staining (Figure 4D,E). Taken together, these results demonstrate that pyroptotic neutrophils release IL‐1β, which, in addition to IL‐18, can suppress corneal re‐epithelialisation after injury.

### 2.4 Faster re‐epithelialisation is associated with expression of Wnt5a and sflt‐1 and reduced corneal neovascularisation

**FIGURE 5:**
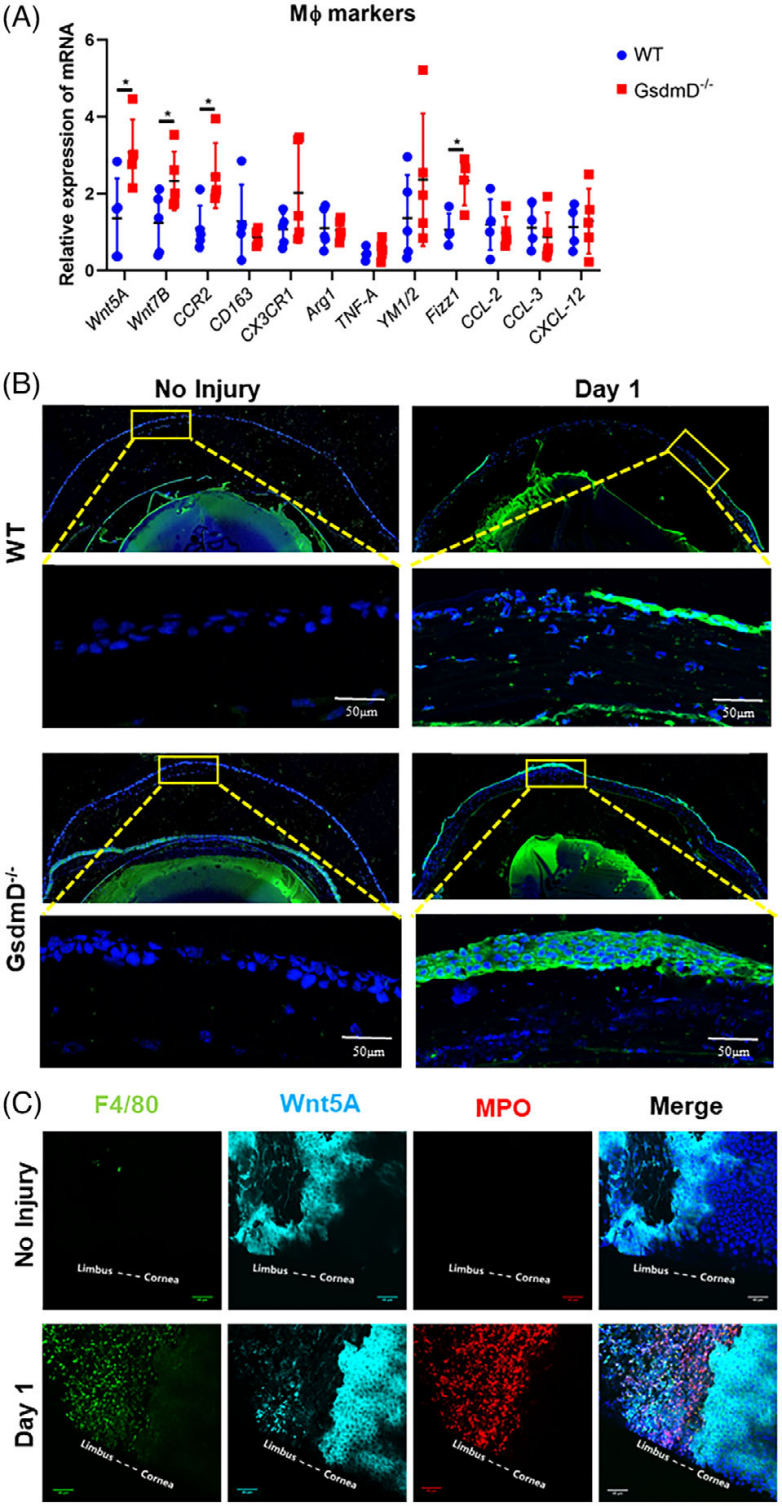
Wnt5A expression is localised to the limbus of uninjured corneas and in newly regenerated corneal epithelium following injury. (A) mRNA expression of several known wound healing markers in corneas derived from WT and *GsdmD^−/−^* mice at 24 h after injury (*n* = 5 for each group). (B) Immunofluorescent staining of mouse eyes shows Wnt5A is highly expressed in the newly differentiated corneal epithelial cells after injury. (C) Flat mount staining of mouse corneas demonstrates that Wnt5A is mainly expressed in limbus before injury. After alkali injury, Wnt5A is expressed in newly generated epithelial cells. Macrophages (F4/80) and neutrophils (MPO) were also observed after injury. Data represent one experiment representative of three independent experiments (A–C).

We next investigated the potential mechanism by which CNV was reduced in *GsdmD^−/−^* corneas. We first tested whether known angiogenesis factors were altered in *GsdmD^−/−^* corneas as compared to WT corneas. Interestingly, although we found that the mRNA expression level of VEGFa dramatically increased in the cornea after injury in both WT and *GsdmD^−/−^* mice, the level of upregulation was similar in both WT and *GsdmD^−/−^* mice (Figure S6A). Thus, we also tested an alternate possibility. Our hypothesis is that corneal re‐epithelialisation primarily hinges on early post‐injury inflammation, whereas vascular growth occurs later and may be more closely associated with macrophages. As previous studies have associated macrophages with CNV,^43^, ^44^ we first examined various markers for macrophages and corneal healing using real‐time RT‐PCR (Figure 5A). We found that expressions of Wnt5A, Wnt7B, CCR2 and Fizz1 were significantly increased in *GsdmD^−/−^* corneas, as compared to those in WT corneas. Among these molecules, Wnt5A has been associated with inhibiting angiogenesis.^45^, ^46^, ^47^, ^48^, ^49^ To confirm our real‐time RT‐PCR finding, we performed Wnt5a IF staining in mouse corneas (Figure 5B). To our surprise, strong Wnt5A signal was found in the newly generated corneal epithelium after injury (Figure 5B). Flat mount staining clearly showed that Wnt5a was mainly expressed in the limbal epithelial compared to the corneal epithelium of the intact ocular surface (Figure 5C). However, after injury, Wnt5A became highly expressed in the newly differentiated epithelial cells (Figure 5C). Further, Wnt5A induction was greater in *GsdmD^−/−^* corneas than that in WT corneas after injury, which coincided with the significantly faster re‐epithelialisation rate that we observed in *GsdmD^−/−^* corneas.

**FIGURE 6:**
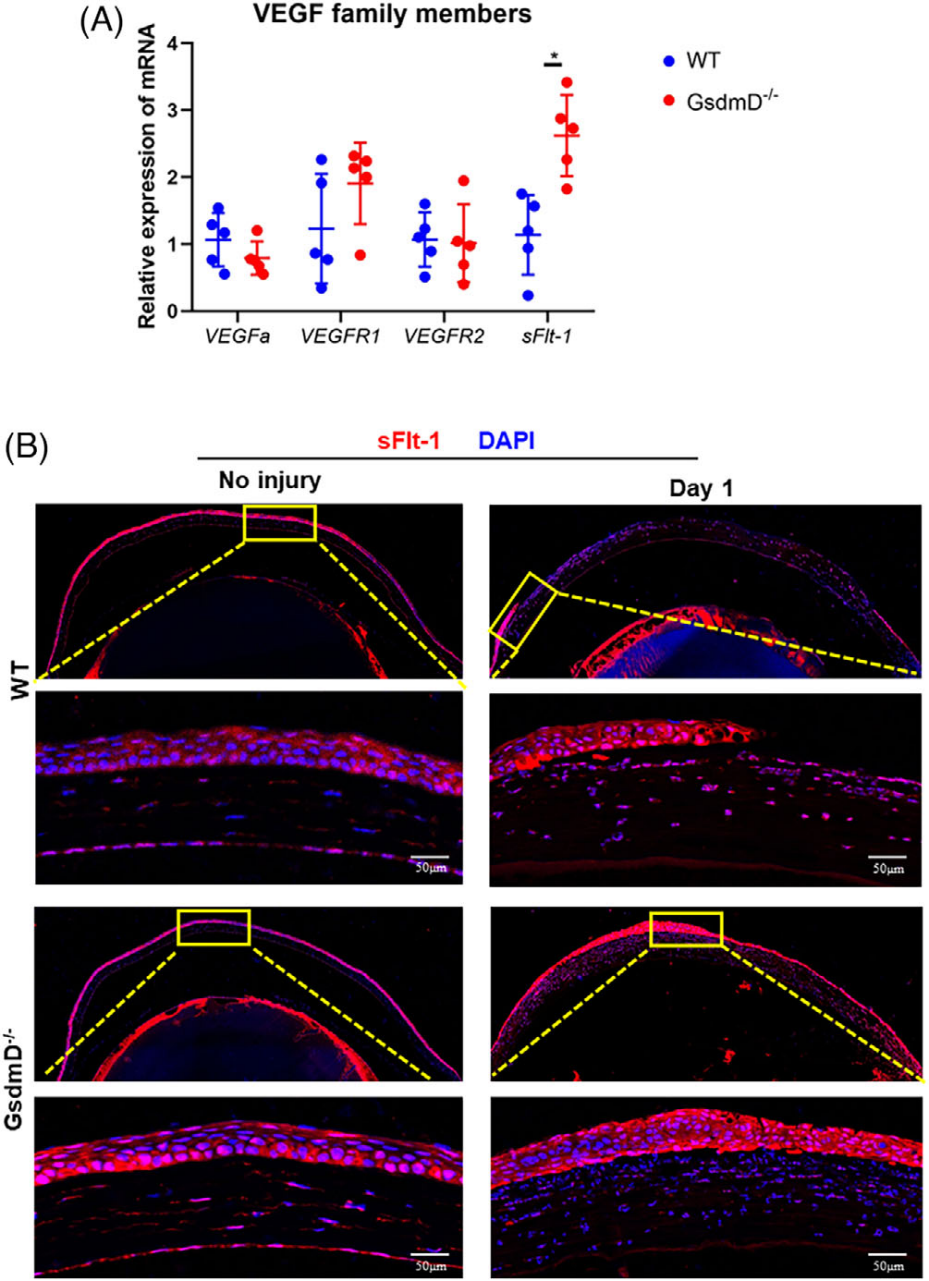
sflt‐1 is highly expressed in newly generated corneal epithelium following injury. (A) mRNA expression of VEGFa, VEGFR1, VEGFR2 and sflt‐1 was quantified using real‐time RT‐PCR from WT and *GsdmD^−/−^* corneas 24 h after injury (*n* = 5 for each group). (B) Expression of sllt‐1, as observed using immunofluorescence, was observed in corneal epithelial and stromal cells before injury and expression of sflt‐1 in epithelial cells was enhanced after corneal injury. Data represent one experiment representative of three independent experiments (A,B).

It is well known that there are several molecules important in maintaining corneal avascularity, one of which is soluble VEGF receptor 1 (sflt‐1).^49^, ^50^, ^51^, ^52^, ^53^ More importantly, previous studies have shown that sflt‐1 is a downstream target of the Wnt5A mediated anti‐angiogenic signalling pathway. Wnt5A‐sflt‐1 axis have been demonstrated to play an anti‐angiogenic role in different tissue and cell types, including placenta, adipose tissue, ischemic muscles, brain and endothelial cells.^45^, ^47^, ^48^, ^54^, ^55^, ^56^, ^57^ We next quantified *sflt‐1* mRNA and found that expression increased in the *GsdmD^−/−^* corneas, as compared to that in WT corneas, after injury (Figure 6A); other angiogenic factors (VEGFa, VEGFR1, VEGFR2) remained similar between the two groups. Consistent with our observation of Wnt5A, IF staining results showed that sflt‐1 was expressed within the cornea, predominantly in the epithelium, and sflt‐1 signal increased after injury (Figures 6B and S6B). Taken together, our studies suggest that sflt‐1, and possibly Wnt5A, signalling in newly generated epithelial cells may play a role in controlling CNV following injury by modulating the local microenvironment through the secretion of sflt‐1, which can bind to and inhibit VEGF‐A. This may explain why injured *GsdmD^−/−^* corneas have faster re‐epithelialisation and develop less CNV when compared to WT corneas.

### 2.5 Bone marrow transplantation from GsdmD^−/−^ to wild‐type mice or neutrophil depletion accelerates corneal wound healing

**FIGURE 7:**
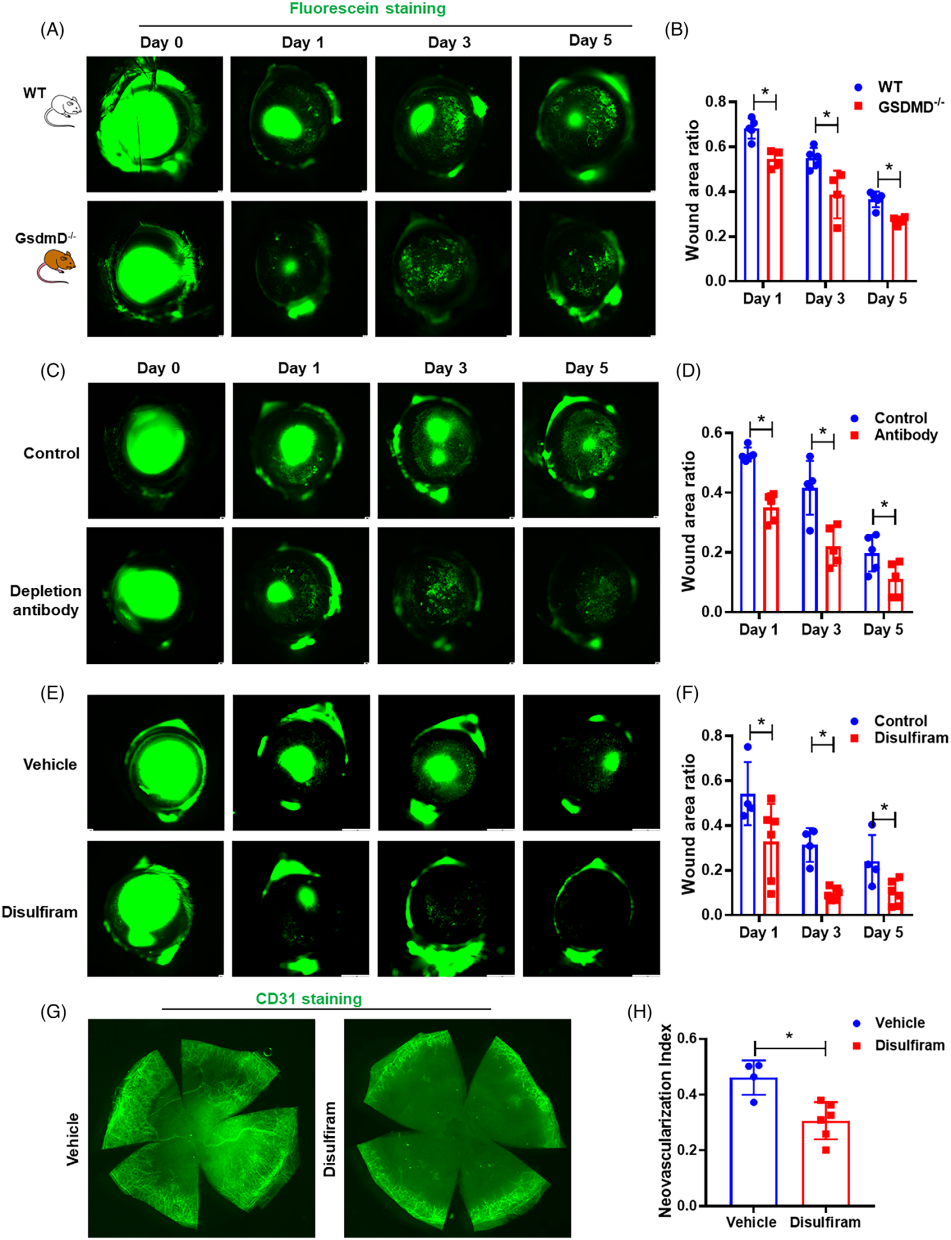
Inhibition of pyroptosis enhances corneal wound healing and inhibits post‐injury corneal neovascularisation. (A) Bone marrow cells from WT and *GsdmD^−/−^* mice were transplanted to irradiated WT mice. Mice that received *GsdmD^−/−^* bone marrow cells had improved re‐epithelialisation (*n* = 5 for WT‐to‐WT mice, *n* = 4 for *GsdmD^−/−^* ‐to‐WT mice) (quantified in B). (C) Dual antibodies treatment to deplete neutrophils (*n* = 5 for each group) or (E) disulfiram treatment to inhibit pyroptosis also promoted corneal re‐epithelialisation (*n* = 6 for disulfiram treated mice and *n* = 4 for sesame oil treated mice) (quantified in D and F). (G) CD31 staining of corneal flat mounts demonstrates that disulfiram treatment could significantly reduce neovascularisation after corneal injury (quantified in H). Data represent one experiment representative of two independent experiments (A–H). WT, Wild type.

In order to test the contribution of neutrophils in corneal wound healing, we first performed a bone marrow transplantation experiment. The following two groups of chimeric mice were generated: WT bone marrow to WT mice (as control), *GsdmD^−/−^* bone marrow to WT mice. The efficiency of bone marrow transplantation was confirmed by Western blot analysis of bone marrow and spleens (Figure S7). Following corneal injury, mice that received *GsdmD^−/−^* bone marrow showed significantly faster wound healing than mice that received WT bone marrow (Figure 7A,B).

Since bone marrow gives rise to many cell types, including monocytes, macrophages, neutrophils and others, the bone marrow transplantation experiments can only partially suggest the potential contribution of neutrophils in corneal wound healing. We next employed a published double antibody‐based depletion strategy^58^ to deplete neutrophils before and after corneal wounding (Figure S8). Consistent with the bone marrow transplantation experiments, mice injected with antibodies showed significantly improved re‐epithelialisation, compared to control animals (Figure 7C,D). Taken together, these results suggest that WT neutrophils inhibit corneal wound healing.

### 2.6 Pyroptosis inhibition promotes corneal wound healing and reduces corneal neovascularisation

While neutrophil pyroptosis plays a detrimental role during corneal wound healing, the initial neutrophil infiltration is critical for removing dead corneal cells and debris, which is part of the healing process. Thus, complete depletion of neutrophils may not be the ideal option to promote normal corneal healing. Disulfiram is an food and drug administration (FDA) approved drug, which has been recently been identified as a pyroptosis inhibitor.^59^ Thus, we tested whether application of disulfiram could be an effective means to promote corneal wound healing. Indeed, as shown in Figure 7E,F, the mice treated with disulfiram showed dramatically accelerated wound healing. Furthermore, the IF staining for CD31 showed that CNV was significantly reduced in the mice receiving disulfiram treatment (Figure 7G,H). To confirm that treatment of disulfiram could inhibit post‐injury pyroptosis, we performed Western blot analysis on corneal samples before and after injury. As expected, disulfiram treatment effectively inhibited pyroptosis after corneal wounding (Figure S9). Thus, our study suggests that disulfiram might be an effective means to promote corneal wound healing and inhibit post‐injury CNV.

### 2.7 Enhanced corneal wound healing and reduced corneal neovascularisation in GsdmD myeloid conditional knockout mice

**FIGURE 8:**
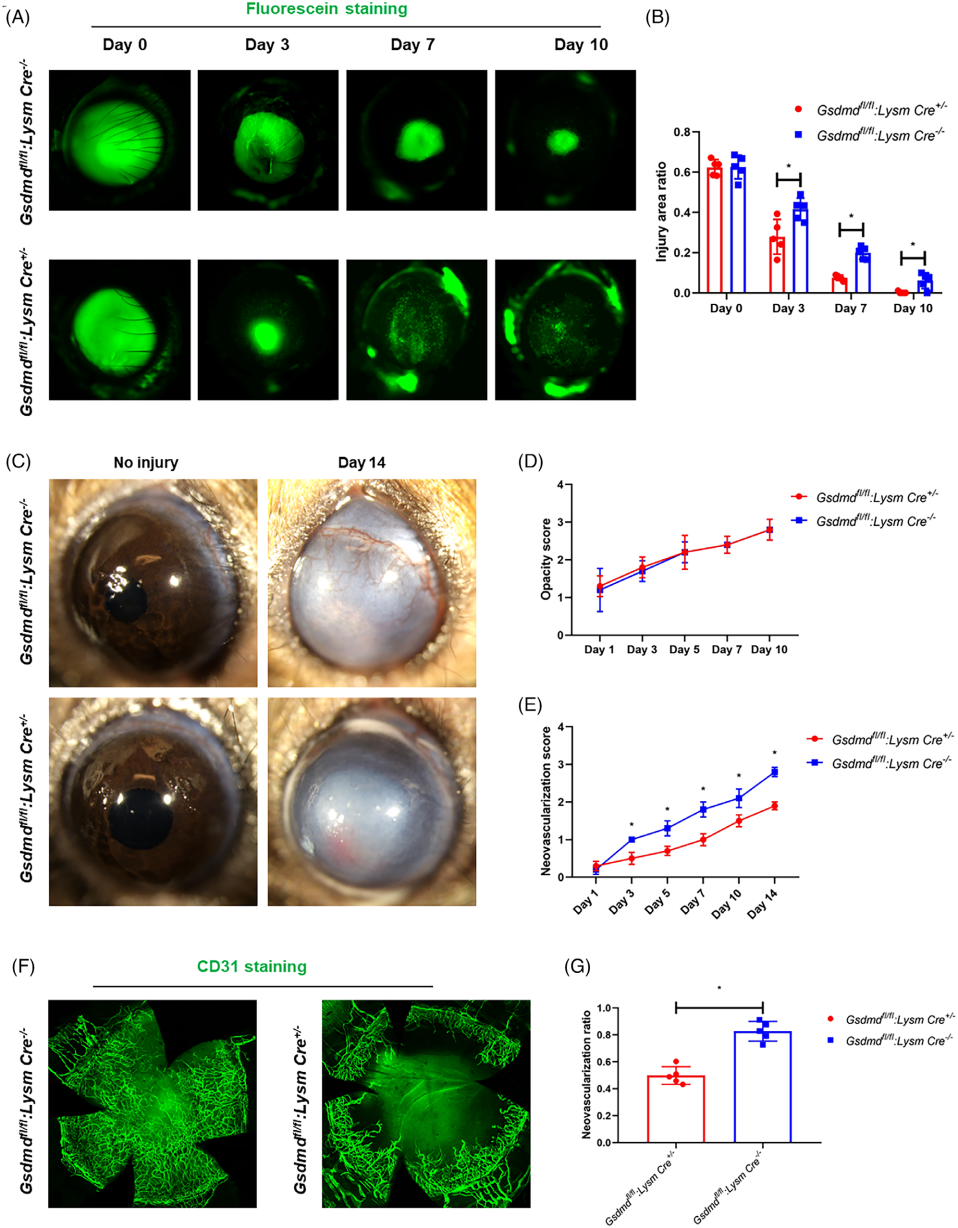
Enhanced corneal wound healing and reduced CNV in GsdmD myeloid conditional knockout mice. Representative images demonstrating fluorescein uptake illustrate enhanced re‐epithelialisation in the corneas of *Gsdmd^fl/fl^*:*Lysm Cre^+/−^* mice compared to those of *Gsdmd^fl/fl^*:*Lysm Cre^−/−^* mice (*n* = 5 for each group). (B) Quantification of fluorescein assay. (C) Representative images of ocular photographs depict corneal fibrosis and neovascularisation at both pre‐injury and 14 days post‐injury. (D) Opacity scores at indicated time points. (E) Neovascularisation scores at indicated time points. (F) Representative images of CD31 staining of flat mount corneas. (G) Quantification of CD31 signal area. Data represent one experiment representative of two independent experiments (A–E). CNV, Corneal neovascularisation.

To further validate the previous research findings, we purchased *Gsdmd flox* mice and bred them with *Lysm Cre* mice to obtain myeloid cell‐specific *Gsdmd* knockout mice (*GsdmD ^fl/fl^:LysM ^Cre/+^*). We isolated neutrophils from the mice and verified *GsdmD* knockout efficiency in neutrophils (Figure S10), and assessed neutrophil viability in vitro through PI staining and flow cytometry. The results indicated that isolated *GsdmD ^fl/fl^:LysM ^Cre/+^* neutrophils had significantly lower rates of death, compared to neutrophils isolated from littermate control mice, at 0 and 24 h after in vitro culture, with no significant differences observed at 48 h (Figure S11). Alkali burn assays revealed a significantly accelerated corneal re‐epithelialisation rate in *GsdmD ^fl/fl^:LysM ^Cre/+^* mice, as compared to littermate controls (Figure 8A,B), with no significant differences observed in corneal fibrosis (Figure 8C,D). Furthermore, significantly less CNV was observed in *GsdmD ^fl/fl^:LysM ^Cre/+^* mice as compared to littermate controls (Figure 8E–G). Interestingly, as observed in flat‐mount staining, there was greater CD31 expression in the corneas of these control mice compared to other control corneas (see Figure 8F vs. Figures 1E and 7G). We reasoned that *first*, the flat‐mount staining for Figure 8 was performed at day 14 after corneal injury, while the flat‐mount staining shown in Figures 1 and 7 were performed at day 10 after corneal injury, *second*, different genetic backgrounds of the mice may influence healing, which requires further investigation. Taken together, these results suggested that neutrophil pyroptosis could be a key regulatory factor for corneal re‐epithelialisation and neovascularisation.

## 3 DISCUSSION

GsdmD has recently been regarded as a negative regulator of innate immunity, where GsdmD deficiency has been shown to delay neutrophil death and enhance host response to bacteria.^36^ The role of neutrophils in the wound healing process is mainly considered beneficial.^60^ Neutrophil infiltration to the wound area is one of the earliest steps of wound healing, enabling phagocytosis of cellular debris and bacteria, allowing for decontamination of the wound.^61^ NETs have also been considered to have dual functions in wound healing. NETosis, originally only considered a defensive mechanism, is now known to induce detrimental effects on tissue physiology, exacerbating pathologies.^62^ As one example, alkali burns induced NET formation in the cornea and impaired epithelial migration.^10^ Although the literature supports the dual functions of neutrophils in the wound healing process, the role of neutrophil pyroptosis in corneal healing has not yet been studied.

In this study, we found that neutrophils infiltrated the injured cornea as early as 3 h and peaked at approximately 24 h after alkali induced injury. We further demonstrate that infiltrating neutrophils quickly undergo pyroptosis. The pyroptotic neutrophils release IL‐1β, which suppressed migration of epithelial cells and compromised epithelial integrity. Consistently, IL‐1β has been reported to delay corneal wound healing by impairing migration and inducing apoptosis of epithelial cells.^63^ Xu et al. also reported that IL‐1β compromised tight junction integrity in bovine mammary epithelial cells.^64^ In support of our study, prior work has demonstrated that inhibition of pyroptosis improves corneal healing following alkali injury.^20^, ^37^ Our study revealed that, along with corneal cells, neutrophils also produce IL‐1β, which may inhibit re‐epithelialisation after corneal wounding.

**FIGURE 9:**
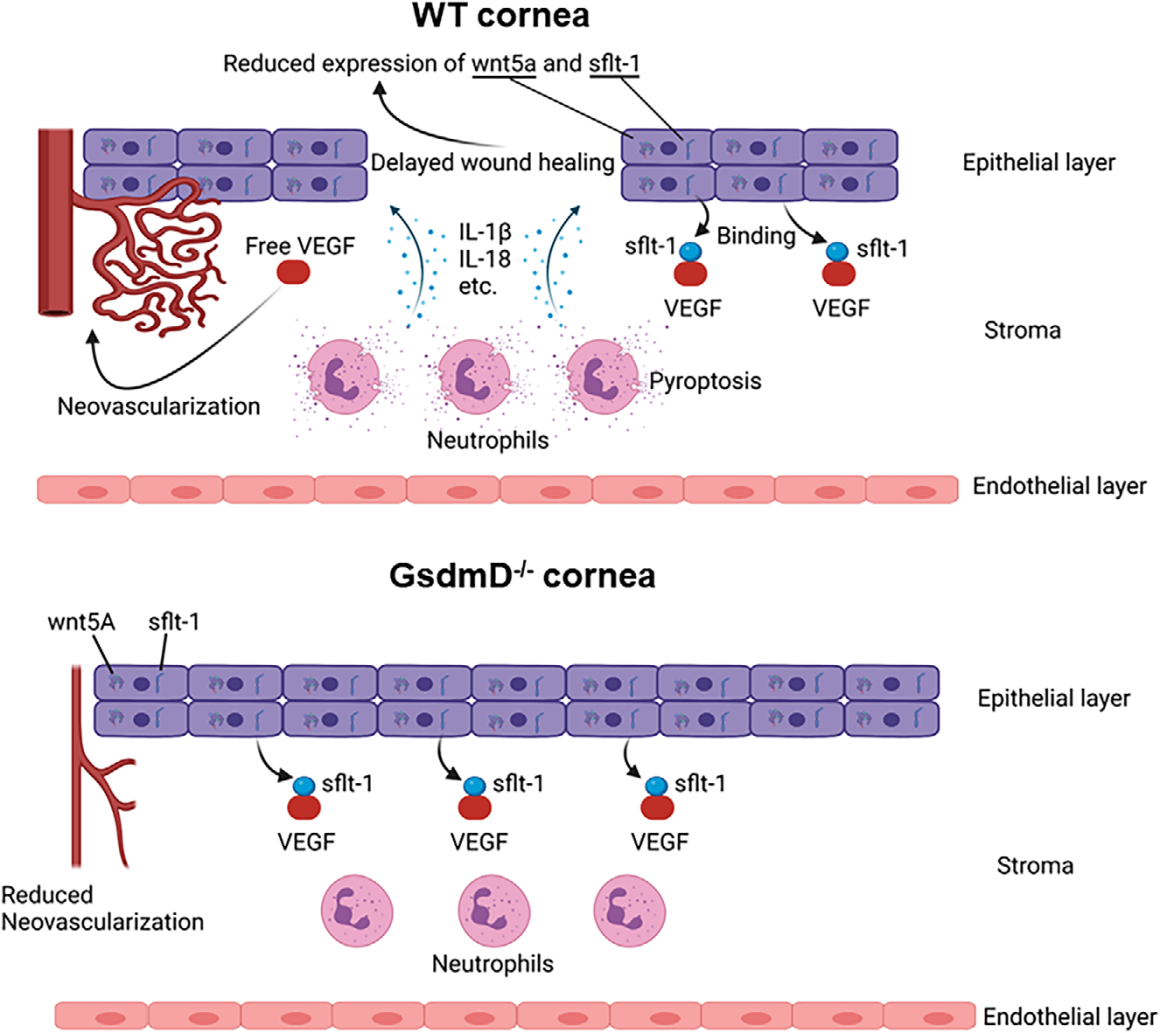
Schematic figure summarising our hypothesised role of neutrophil pyroptosis in regulating corneal wound healing and post‐injury neovascularisation. Neutrophil pyroptosis reduces corneal epithelial cell migration and healing rates. Subsequent loss of the corneal epithelium results in reduced expression of sflt‐1 and Wnt5a which may promote neovascularisation. Inhibition of neutrophil pyroptosis improves corneal re‐epithelialisation which restores expression of anti‐angiotic factors, such as Wnt5a and sflt‐1, to improve healing and reduce CNV. CNV, Corneal neovascularization.

During normal homeostasis and healing, progenitor cells, derived from stem cells located within the limbal niche, repopulate the corneal epithelium.^65^, ^66^ This distinct niche, composed of multiple cell types, is critical to the maintenance of limbal stem cells and to the avascularity of the cornea.^66^ During wound healing, limbal stem cells differentiate into corneal epithelium and these differentiated corneal epithelial cells express essential anti‐angiotic factors to maintain corneal angiogenic privilege.^51^ One such corneal soluble anti‐angiotic factor is sflt‐1, which is expressed at low levels by normal corneal epithelium, where it acts as an antagonist to the VEGF action.^50^ In our study, we confirm that sflt‐1 is expressed predominantly in the corneal epithelium. After alkali injury, while mRNA expression of VEGFa was increased nearly 10‐fold in the injured cornea at 24 h (Figure S6), the sflt‐1 expression did not concurrently increase, likely due to loss of the corneal epithelium, resulting in a possible imbalance between VEGFa and sflt‐1 expression. We hypothesise that this injury induced imbalance between VEGFa and sflt‐1 might be one reason post‐injury CNV is observed. Therefore, the rapid repair of the corneal epithelium may play a key role in restoring the balance between VEGFa and sflt‐1 and reducing subsequent CNV (summarised in Figure 9).

In addition to sflt‐1, our data demonstrate that the newly generated corneal epithelial cells express high levels of Wnt5A, which has been reported as an angiogenesis inhibitor in the retina by upregulating sflt‐1.^49^ Accumulating evidence indicates that the Wnt5a‐sflt‐1 pathway can inhibit neovascularisation in different tissues, including placenta, adipose tissue, ischemic muscles and brain. ^45^, ^47^, ^48^, ^54^, ^55^, ^56^, ^57^ Not directly tested in the current work, it is possible that Wnt5a behaves similarly in the cornea, as a non‐canonical regulator of sflt‐1. Alternatively, others have shown that Wnt/β‐catenin signalling is important for limbal epithelial stem cell survival and propagation in vitro and that Wnt‐5a improves ex vivo diabetic corneal healing.^67^, ^68^, ^69^, ^70^ Furthermore, endothelial Wnt5A has been shown to promote retinal and tumour angiogenesis.^71^ Therefore, the effects of Wnt5A might vary in different tissues and cells.

Disulfiram is an FDA approved drug, which has been reported to be a pyroptosis inhibitor.^72^ We found that disulfiram cannot only inhibit the release of mature IL‐1β, but the drug can also inhibit the cleavage of caspase 1 and GsdmD, needed for pyroptosis activation. It should be noted that disulfiram has been reported to have multiple molecular targets and various tissue specific and systemic effects.^73^ Of note, disulfiram can reduce NF‐κB‐mediated inflammation and modulate reactive oxygen species formation,^74^ both of which, may play important roles in corneal healing and may contribute to off‐target effects in this study. While investigation of alternate mechanisms of action are warranted, we demonstrate that mice treated with disulfiram have improved healing outcomes compared to controls and as such, disulfiram could be an effective treatment to promote corneal healing and inhibit CNV post‐injury.

Interestingly, although the results of this study indicate that inhibition of GsdmD improved re‐epithelialisation and reduced CNV, the degree of corneal fibrosis did not significantly differ. Corneal fibrosis is a significant cause of blindness indicating that additional strategies, beyond GsdmD inhibition, are likely needed to restore corneal clarity. Macrophages are a major source of transforming growth factor‐β in wounded tissues.^75^ In this study, macrophages were observed within the limbus and less so in the cornea; however, expression of the macrophage marker F4/80 was only evaluated at early times when fibrosis was not evident. It is possible that additional immune cells, such as macrophages or dendritic cells, were less affected by GsdmD inhibition and continued to promote tissue fibrosis beyond the initial time points evaluated here.

There are several limitations to this study. Our data demonstrate that Wnt5a induction and sflt‐1 expression were greater in *GsdmD^−/−^* corneas, compared to controls, and this increase corresponded to changes in re‐epithelialisation and CNV. Although this work represents an association between reduced neutrophil pyroptosis and improved corneal healing, a direct cause has not been proven. Additional experiments could include bone chimera reconstitution of neutrophils from wild‐type to *GsdmD^−/−^* mice to determine if the reciprocal outcome (i.e., reduced Wnt5a and sflt‐1 expression) was observed following injury. Further, this work only evaluated one type of injury response. It would be interesting to compare the current data to other injury or disease model systems, such as corneal infections or dry eye. Since IL‐18 and IL‐1β are two major effector molecules of neutrophil pyroptosis, we simultaneously investigated the effects of these two cytokines on corneal epithelial cell wound healing. However, we indeed lack direct evidence to support the release of IL‐18 by neutrophils after corneal injury, further studies are still needed. Other cytokines such as TNF‐a, IL‐6, and IL‐12, which are also produced by infiltrating activated neutrophils and macrophages should also be examined in the future study to gain a more comprehensive understanding of the inflammatory response. Additional experiments such as RNA sequencing could also add insight into key molecular pathways involving neutrophil pyroptosis and corneal healing.

Taken together, our results reveal the important role of neutrophil pyroptosis during the cornea wound healing process. The inhibition of neutrophil pyroptosis after injury could be a potential clinical therapeutic to promote corneal healing and inhibit CNV.

## 4 MATERIALS AND METHODS

### 4.1 Animals

For the mouse corneal chemical injury model, the mice were treated under anaesthesia. All animals received topical antibiotics. Topical and systemic analgesics were provided for at least 72 h. To induce corneal injury, a 1.5 mm filter paper soaked in 1 M NaOH was applied to the axial cornea for 30 s. The cornea was then rinsed with 20 mL of saline, dry the area with sterile cotton gauze. After the cornea was thoroughly rinsed, fluorescein stain was applied to the cornea to verify corneal ulceration.

GsdmD^−/−^ mice were kindly provided by Dr. Vishva M. Dixit from Genentech, Inc. The *GsdmD* genotyping primers are: WT: Forward: GTAGTGCTGTGGTTGCTGGGATT, Reverse: GAAATTTTCCCTTCTCCCATGCCTGACGAC. Mutant: Forward: GATGGGAACATTCAGGGCAGAGTGATGCTT, Reverse: CGGCTGCTCAAGGTAAGG.

The knockout band size is 202 bp, while the WT band size is 273 bp. C57Bl/6J mice were obtained from the Jackson laboratory.

*Gsdmd Flox* mice (Cat. No. NM‐CKO‐190060) were purchased from Shanghai Model Organisms Center, Inc. *LysM cre* mice (JAX stock #004781) were purchased from Jax laboratories.^76^

### 4.2 Clinical evaluation

The clinical opacity scores and clinical vascularisation scores were determined by single observer, masked to the animal grouping, using a modified Hackett–McDonald scoring system. The evaluations conducted immediately following cornea injury and subsequently every 24 h. For specific scoring criteria, please refer to our previous publication.^77^ Size of the cornea wound was verified using fluorescein.

The mouse eye images in the aforementioned examination were captured using the Leica THUNDER Imaging System. Images of wound fluorescein were quantified by using ImageJ software.

### 4.3 Preparation of recombinant IL‐1β and IL‐18 proteins

The coding sequences of human pro‐IL‐18 and pro‐IL‐1β were cloned into the PET28a vector, where an *N*‐terminal 6xHis tag is added. The purification procedure is similar to the published protocol with minor modifications.^78^ Briefly, the respective plasmid was transformed into BL21 (DE31) chemically competent cells. A single colony was picked to grow in LB media at 37°C until OD600 researched .6, and .5 mM IPTG was used for overnight induction at 16°C. Harvested cells were collected by centrifugation, followed by resuspension in 50 mM Hepes (pH 7.5), 5 mM MgCl_2_, 1 mM PMSF, .5 mg/mL lysozyme and 50−100 U DNase I. After sonication, the cell lysates were centrifuged. The supernatant was then applied to a Ni‐NTA affinity chromatography, where buffer A contained 50 mM Hepes (pH 7.5) and Buffer B contained an extra 500 mM imidazole. Fractions with the highest purities were pooled and applied to a diethylaminoethyl‐cellulose (DEAE) ion exchange chromatography, where buffer A’ contained 50 mM Hepes (pH 7.5) and Buffer B’ contained an extra 500 mM NaCl. As judged by SDS‐PAGE, fractions with > 95% purity were pooled and dialysed in 50 mM Hepes (pH 7.5) and 100 mM NaCl.

Recombinant human caspase‐1 was purchased from Enzo Life Sciences (ALX‐201‐056). The cleavage of pro‐IL‐18 or pro‐IL‐1β was performed at 37°C in phosphate‐buffered saline (PBS) buffer containing 2 mM dithiothreitol. The concentration of protein used for the cell treatment is 20 ng/mL.

### 4.4 Drug or antibody treatment

For disulfiram treatment of mice, disulfiram was dissolved in sesame oil (12.5 mg/mL). Mice were injected16 h before injury through intraperitoneal injection (50 mg/kg); injection continued daily for the duration of the experiment. For Gr‐1 antibody treatment, mice were injected 16 h and immediately before injury through intraperitoneal injection (100 µg/mouse). For double antibody‐based depletion strategy treated mice, both anti‐Ly6G (clone 1A8, #BP0075‐1) and anti‐rat kappa light chain ((clone MAR 18.5, #BE0122) antibodies were injected starting 2 days before injury. Anti‐Ly6G antibody was administered daily, whereas Anti‐rat kappa light chain antibody was injected every other day.^58^

### 4.5 Bone marrow Chimeras

Mice were treated by split‐dose irradiation. First with a 350 rad dose, and second by a 950 rad dose 24 h later. Four hours after the second dose of irradiation, mice were injected intravenously with 5×10^6^ flushed bone marrow cells from the femur and tibia of donor mice. The dosage and method of irradiation were based on this publication.^79^ The following two groups of chimeric mice were generated: WT to WT, Gsdmd^−/−^ to WT. The mice were underwent corneal injury 6 weeks after bone marrow transfer and the reconstitution efficiency were determined by Western blot after sacrificing the mice.

### 4.6 Cell lines and treatments

Mouse primary corneal epithelial cells (mCECs) were purchased from Cell Biologics, Inc. (Chicago, IL, Cat. No. C57‐6048). mCECs were cultured in mouse epithelial cell medium (Cell Biologics, Inc., Cat. No. M6621).

### 4.7 Cell scratch wound healing assay

To determine the cell migration rate, an in vitro scratch wounding was performed using mCECs. Cells were seeded onto the detection plate after recovery and stable passaging. It typically took 24−48 h for the cells to reach confluence, and the experiment start time was determined based on observations. Cells were treated with dissected corneas or cytokines when they reached confluence, were subsequently scratched with a micropipette tip, and incubated for up to 12 h. Images were taken at 0, 3, 6, and 9 h after the scratch wound. Images of wound closure were quantified by using ImageJ software.

For co‐culture experiments, mCECs were cultured in 24‐well plates and allowed to reach confluence. From prepared euthanised mice, the corneas were immediately removed and transferred into the corresponding wells. Primary neutrophils were isolated and immediately seeded into Transwell inserts (.4 µm polyester membrane). The mCEC subsequently underwent scratch formation using a micropipette tip; image acquisition and analysis were performed as described above.

### 4.8 Transepithelial electrical resistance measurement

mCEC were seeded in transwell inserts (2×10^5^ per well) and allowed to become confluent. For the protein treatment, proteins were added into the culture medium 16 and 4 h before measurement; the EVOM2 instrument was used to measure the TEER.

For the co‐culture experiments, mCEC were seeded in the transwell inserts and allowed to become confluent. The isolated primary neutrophils were then seeded into 24‐well plates and the EVOM2 instrument was used to measure the TEER at 24 h.

### 4.9 Mouse primary neutrophil isolation

Bone marrow samples were obtained as previously described.^80^ Briefly, lower limbs were collected from euthanised mice. All attached soft tissue was removed to fully expose the femurs and tibias. A 25G needle was inserted into hollow of the bone and pulse flush buffer (2% fetal bovine serum in PBS) used to flush marrow into a 15 mL sterile centrifuge tube. The solution was then filtered with a 70−100 µm filter. The solution was centrifuged at 3 000×*g* for 3 min, and the bone marrow cells were resuspended in red blood cell lysis buffer for 5 min.

Separation of neutrophils using density gradient centrifugation was performed as previously described.^80^ The bone marrow cells were resuspended in 1 mL of ice‐cold sterile PBS. Using a 15 mL conical tube, 3 mL of Histopaque 1119 was first added followed by an overlay of 3 mL of Histopaque 1077. The bone marrow cell suspension was then overlaid on the Histopaque 1077. The samples were centrifuged for 30 min at 1 000×*g* at 25°C. Neutrophils were then at the interface of the Histopaque 1119 and Histopaque 1077 layers. The collected neutrophils were washed twice with culture medium and centrifuged at 1 000×*g* for 3 min.

### 4.10 Flow cytometry

The prepared cells were first stained with PI following previously published methods,^81^ and after staining, they were analysed on Guava easyCyte Instrument. Cell debris was excluded based on forward scatter and side scatter parameters. The range of PI‐positive cells was determined using the same cell population without PI staining as a control. Subsequently, the percentage of PI‐positive cells was calculated.

### 4.11 Western blot

Protein lysates derived from indicated samples were separated by SDS‐PAGE. The antibodies used in this study were: anti‐GsdmD antibody (Abcam, Cat. No. ab219800); anti‐IL‐1β antibody (Abcam, Cat. No. ab4722); anti‐human caspase 1 antibody (Cell Signaling Tech, Cat. No. #3866); anti‐mouse caspase 1 antibody (BioLegend, Cat. No. 645102); anti‐MPO antibody (R&D systems, Cat. No. AF3667); anti‐GAPDH antibody (Cell Signaling Tech, Cat. No. 2118s); anti‐NLRP3 antibody (Cell Signaling Tech, Cat. No. 15101S). Secondary antibodies, anti‐mouse, anti‐rabbit, anti‐rat, or anti‐donkey IgG HRP conjugated, were applied at 1:5 000 dilution.

### 4.12 Quantitative RT‐PCR analysis

Total RNA was extracted from dissected corneas by using TRIzol reagent (Invitrogen, CA, Cat. No. 15596026). Around 500 µµg of total isolated RNA was reverse transcribed into DNA (Thermo Scientific, Cat. No. 1651). The DNA products were quantified by real‐time PCR with SYBR Green Real‐Time PCR mix (Thermo Scientific, Cat. No. A25778). All primer sequences used in the current study are summarised in Table S1.

### 4.13 Histopathology and immunofluorescent staining

Dissected eye from mice were fixed in 4% paraformaldehyde (PFA) for 24 to 48 h at 4°C. The eyes were processed as follows: 2 h 50% ethanol, 1.5 h 70% ethanol, 1 h 80% ethanol, 1 h 90% ethanol, 30 min 95% ethanol, 30 min 95% ethanol, 15 min 100% ethanol, 15 min 100% ethanol, 15 min xylene, 15 min xylene, 30 min paraffin wax, 1 h paraffin wax, 1 h paraffin wax. After embedding, 5 µm thick paraffin sections were cut and stained with H&E or IF staining. The procedures for IF staining of paraffin embedded samples were: deparaffinise/hydrate sections: xyleneI: 7 min, xylene II: 7 min, 100% ethanol I: 5 min, 100% ethanol II: 5 min, 95% ethanol: 3 min, 70% ethanol: 3 min, 50% ethanol: 3 min, ddH_2_O I: 3 min, ddH_2_O II: 3 min, Antigen retrieval: tris‐EDTA buffer (10 mM Tris, 1 mM EDTA, pH 9.0). Slides and a microwavable vessel were placed inside the microwave, set to full power and wait until the solution comes to a boil. Continue to boil for 15 min. Slowly, cool to room temperature then wash in PBS 3 times/3 min. Slides were then blocked in 3% bovine serum albumin (BSA) in phosphate‐buffered saline with tween 20 (PBST) for 1 h at room temperature. Blocking solution was removed and primary antibody diluted in tris‐buffered saline with tween 20 (TBST) with 1% BSA added to each section. Slides were incubated overnight at 4°C. Slides were then washed in PBST three times for 5 min each. Fluorescently labelled secondary antibody, diluted in TBST with 1% BSA was then added to each slide and incubated for 60 min at room temperature. Slides were then washed in PBST three times for 5 min each. Slides were mounted with coverslips and mounting medium containing DAPI.

For flat mount staining, eyes were fixed in 4% PFA at 4°C for 24–48 h, then transferred to PBS. Corneas were dissected, washed with PBS for 3 times (10 min for each wash), blocked in blocking buffer (3% BSA and 0.5% Triton X‐100 in PBS) for 2 h at RT, before application of primary antibody (1:100 diluted in blocking buffer) and incubation at 4°C overnight. Subsequently, corneas were washed 6 times with washing buffer (0.5% Triton X‐100 in PBS) for 1 h each time at RT, and secondary antibodies (1:500 diluted in blocking buffer) applied and incubated at 4°C overnight. Finally, the corneas were washed with PBS three times at RT for 1 h each time; four cuts were made in the corneas to facilitate mounting (DAPI Fluoromount‐G, SouthernBiotech, Cat. No. 0100−20) on slides.

The primary antibodies used for IF staining in this study were anti‐Cleaved GsdmD (N‐terminal) antibody (Cell Signaling Tech, Cat. No. #50928, Discontinued); anti‐IL‐1β antibody (Abcam, Cat. No. ab4722); anti‐MPO antibody (R&D systems, Cat. No. AF3667); FITC anti F4/80 antibody (Biolegned, Cat. No. 123108); anti VEGF Receptor‐1 (Soluble) sflt‐1 antibody (Invitrogen, Cat. No. 36−1100); anti Wnt5A antibody (Thermo Scientific, Cat. No. PA5117496); anti‐CD31 (BD Biosciences, Cat. No. 550274). Secondary antibodies, Alexa Fluor 546 Donkey anti Rabbit IgG (Life Technologies, Cat. No. A10040), Alexa Fluor 647 Donkey anti Goat IgG (Life Technologies, Cat. No. A21447), Alexa Fluro 647 Goat anti Rabbit IgG (Invitrogen, Cat. No. A21244), Alexa, Fluro 488 Goat anti Rat IgG (Invitrogen, Cat. No. A11006) were applied at 1:500 dilution.

The immunofluorescence staining images were captured using the LSM780 laser confocal microscope from ZEISS and the R1 laser confocal microscope from Nikon.

### 4.14 Statistical analysis

All data are presented as mean ± standard deviation (SD) or standard error of the mean (SEM). Groups were compared by Student's *t*‐test and analysis of variance for repeated measures. For comparing the means of three or more groups, ANOVA analysis was performed. A value of *p* < 0.05 was considered significant.

## AUTHOR CONTRIBUTIONS

Hua Zhu, Heather L. Chandler, and Peng Chen conceived the study. Hua Zhu, Peng Chen Heather L. Chandler and Haitao Wen designed the experiments. Peng Chen, Zhentao Zhang, Lilian Sakai, Yanping Xu, Shanzhi Wang, Kyung Eun Lee, Bingchuan Geng, Jongsoo Kim, Bo Zhao, and Qiang Wang performed research and data analyses. Haitao Wen provided valuable suggestions to the study. Hua Zhu, Peng Chen and Heather L. Chandler wrote the manuscript.

## CONFLICT OF INTEREST STATEMENT

The authors declare no conflict of interests.

## ETHICAL APPROVAL

All animal care and usage followed NIH guidelines and were in accordance with the ARVO Statement for the Use of Animals in Ophthalmic and Vision Research. Rodent studies received IACUC approval by The Ohio State University (IUCAC Protocol 2016A00000017‐R2).

## Supporting information

Supporting Information

Supporting Information
