# Supplementary material for: Neutrophil pyroptosis regulates corneal wound healing and post‐injury neovascularisation
Source: Clin Transl Med. 2024 Nov 4;14(11):e1762. doi: 10.1002/ctm2.1762 (PMC11534482; doi:10.1002/ctm2.1762)
Supplement: Supplementary file 2 — Supporting Information [file CTM2-14-e1762-s001.docx]

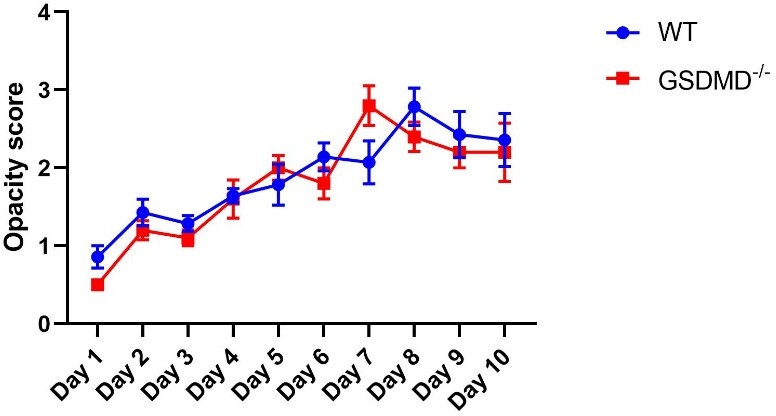


Supplemental Figure S1. Clinical opacity scores show no difference between

***GsdmD-/-* and WT corneas after injury.**


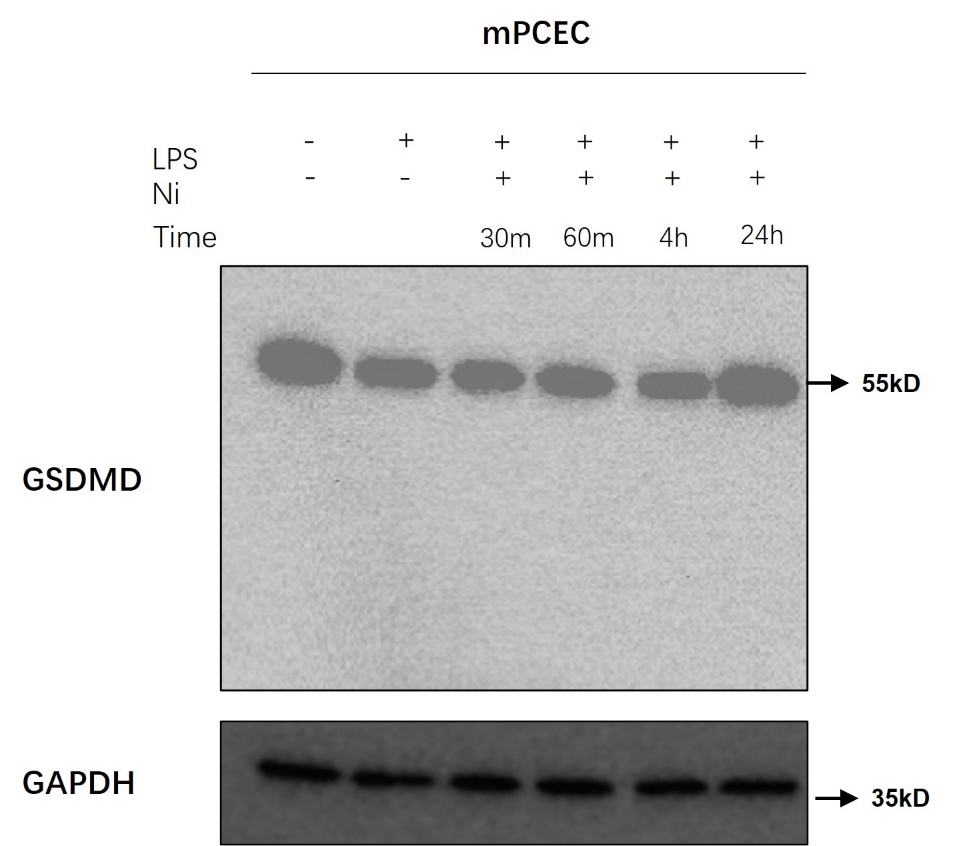


Supplemental Figure S2. Combination of LPS and Nigericin fail to induce pyroptosis in mouse primary cultured epithelial cells (mPCEC).


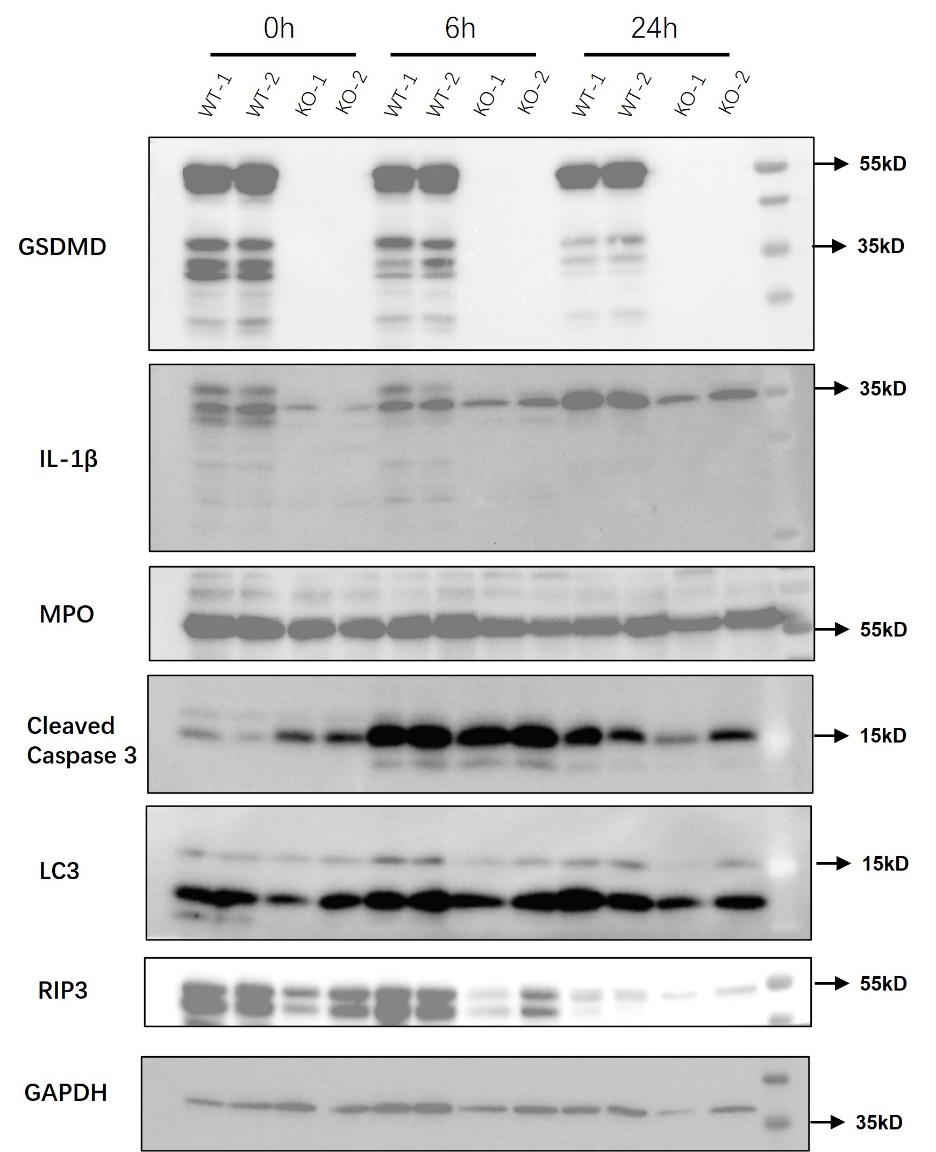


Supplemental Figure S3. Primary cultured neutrophils first undergo pyroptosis and release IL-1β, followed by apoptosis (cleaved caspase 3).


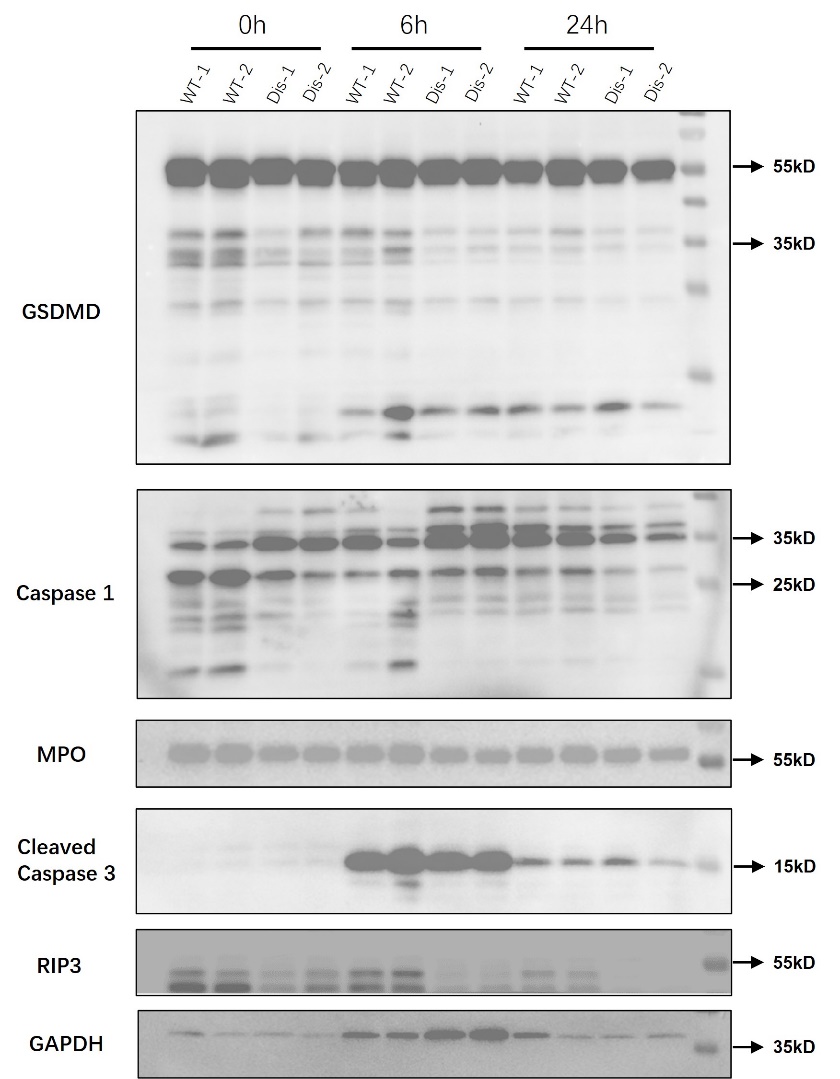


Supplemental Figure S4. Treatment of disulfiram inhibits cleavage of GsdmD but not cleavage of Caspase-3 in primary cultured neutrophil.


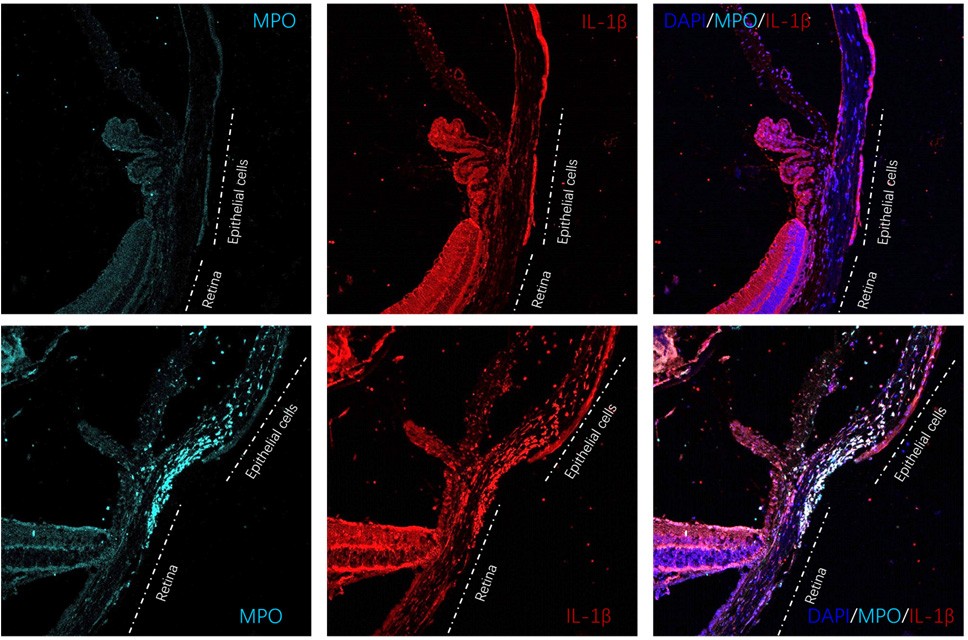
**No injury**

**Day 1**

Supplemental Figure S5. IF staining showed increased IL-1β signal after injury and this signal mainly co-localized with MPO in the cornea 1 day after injury.

**A**


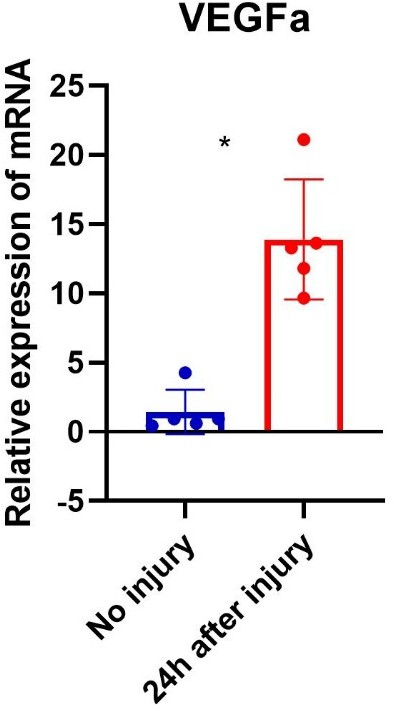

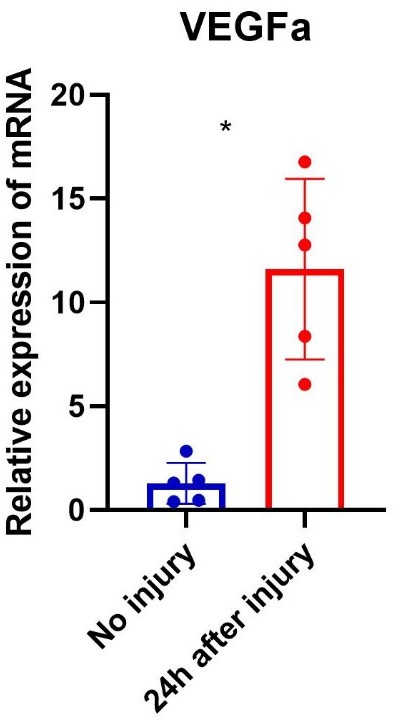


**B**


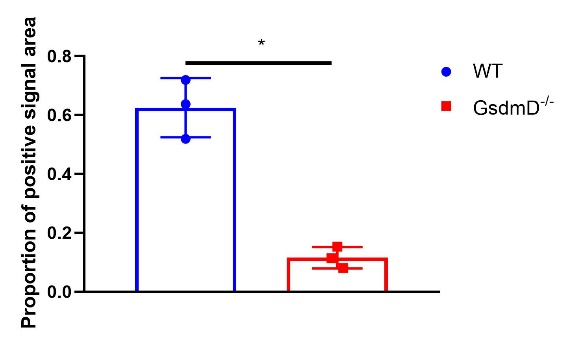


**WT GsdmD-/-**

Supplemental Figure S6. (A) Realtime RT-PCR showed upregulation of VEGFa in both WT and *GsdmD-/-* corneas in similar level. (B) Quantification of sflt1 in injured cornea.


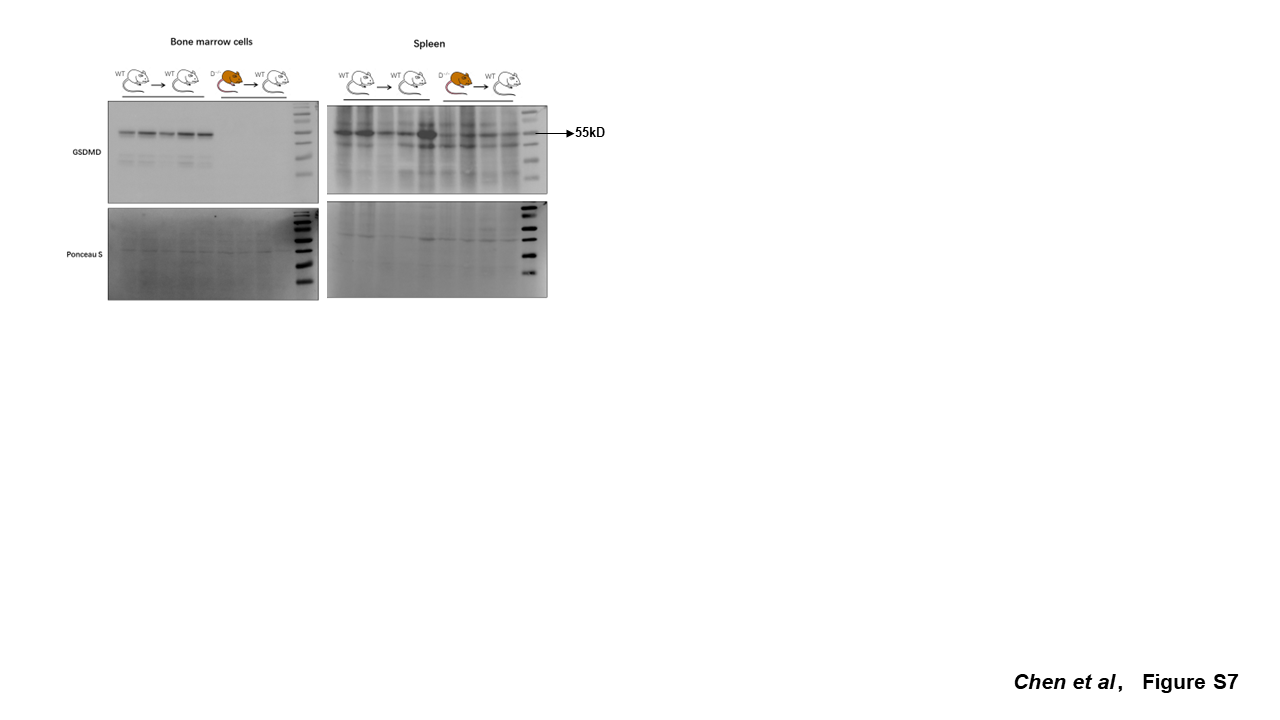


Supplemental Figure S7. The expression of GsdmD in the spleen and bone marrow cells flushed from WT mice that received bone marrow transplantation from WT and *GsdmD-/-* mice, respectively. Ponceau S were used as internal control.


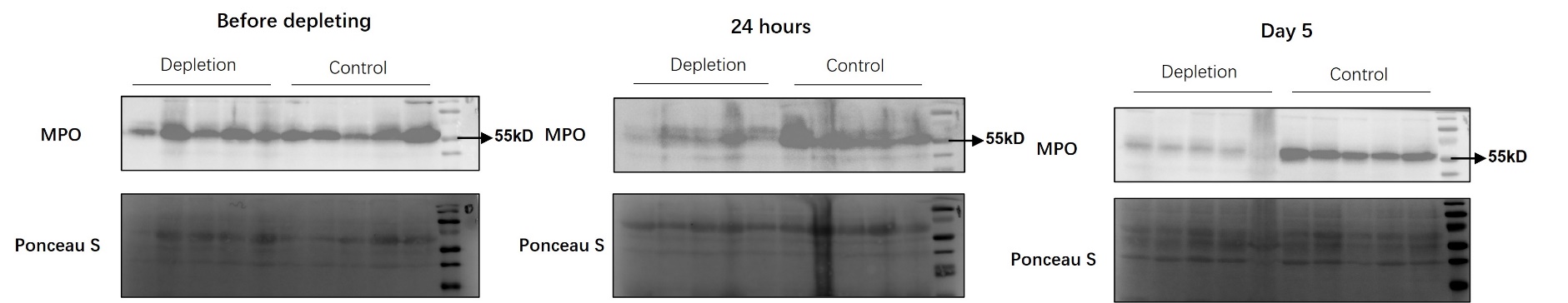


Supplemental Figure S8. Western blotting analysis confirms depletion of neutrophils after antibodies injection at indicated time points.


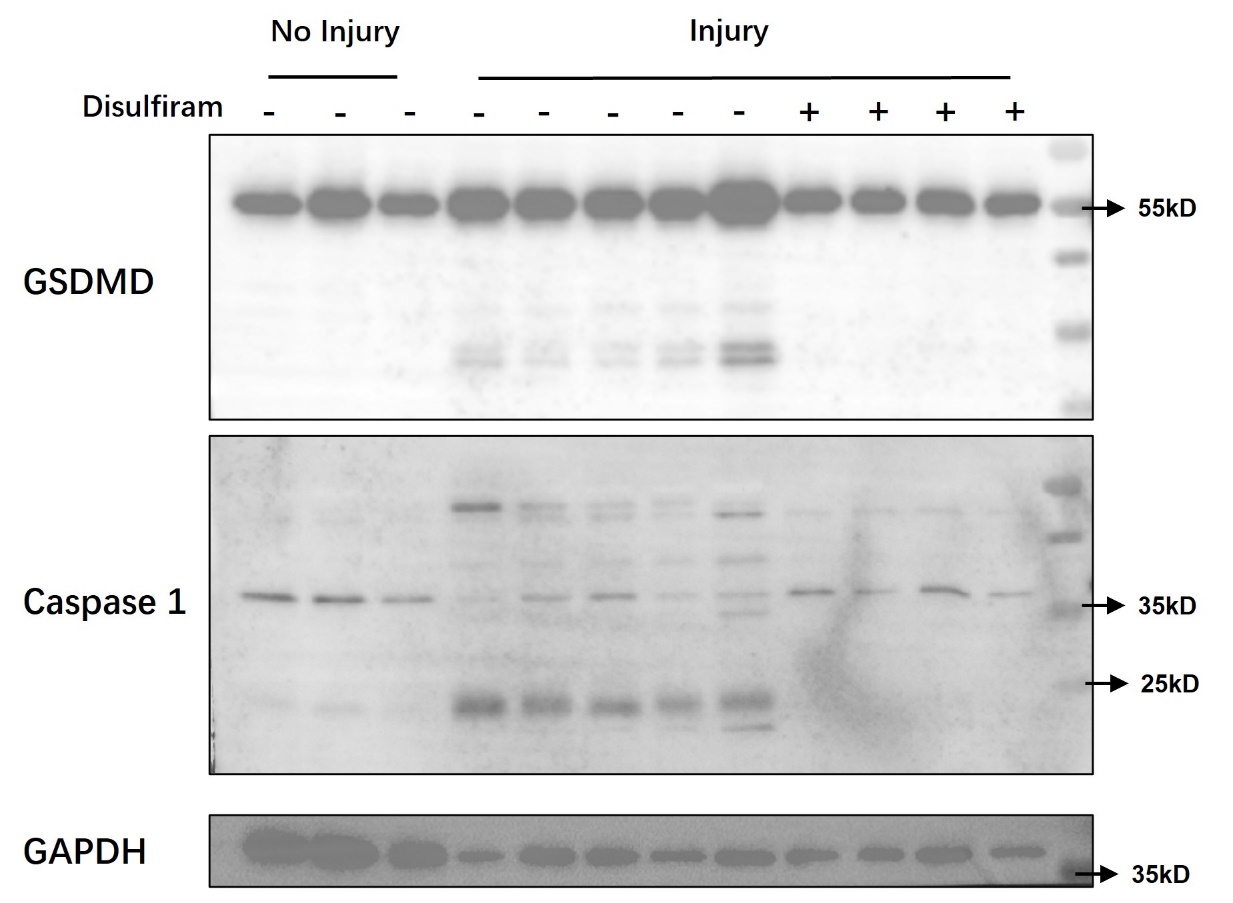


Supplemental Figure S9. Treatment of disulfiram could suppress cleavage of both GsdmD and Caspase-1.


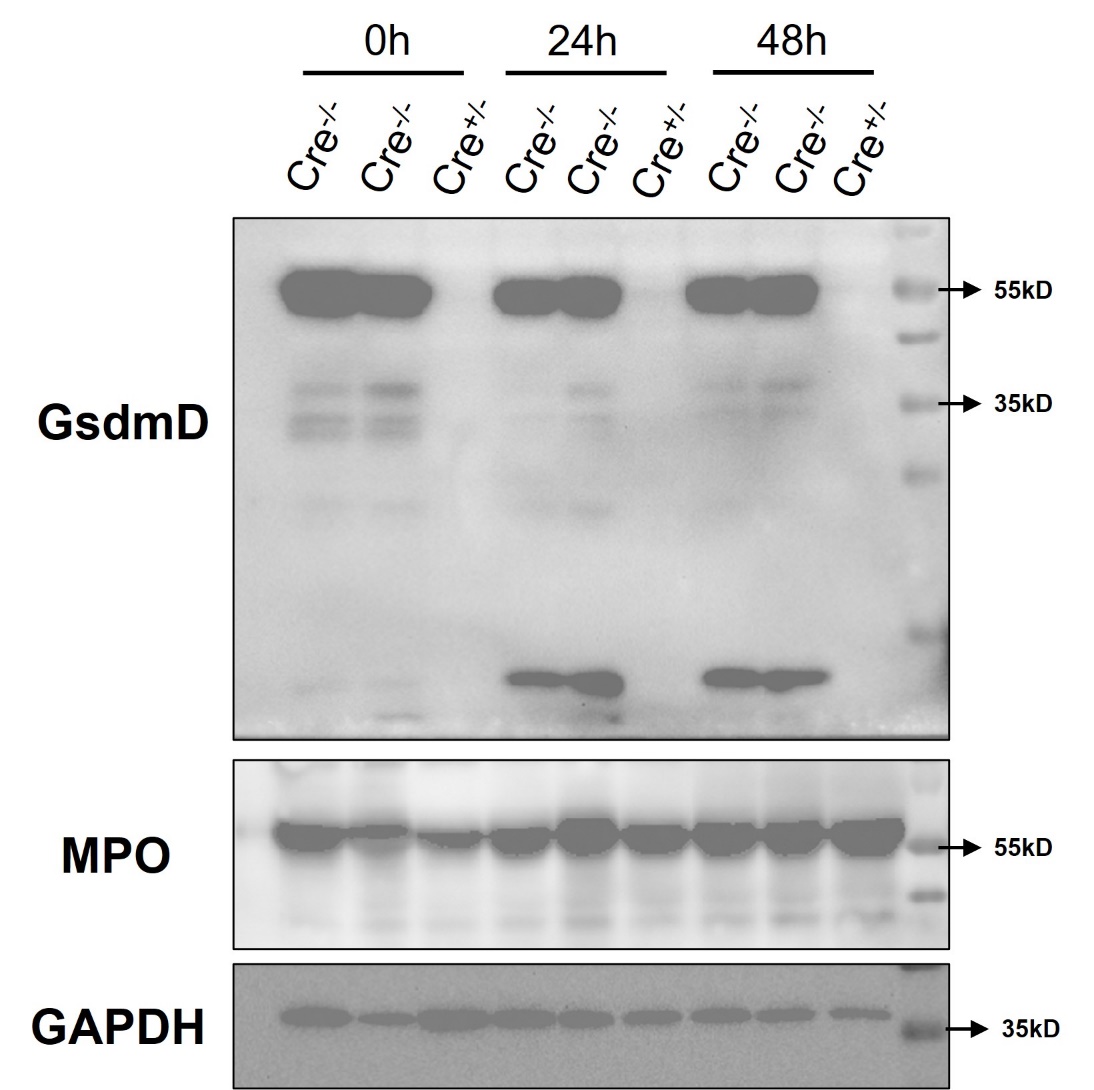


Supplemental Figure S10. The expression of GsdmD, MPO and GAPDH in the isolated bone marrow derived neutrophils cultured at different time points from *Gsdmd^fl/fl^:Lysm^Cre+/-^* mice (Cre+/-) and littermate controls (Cre-/-).


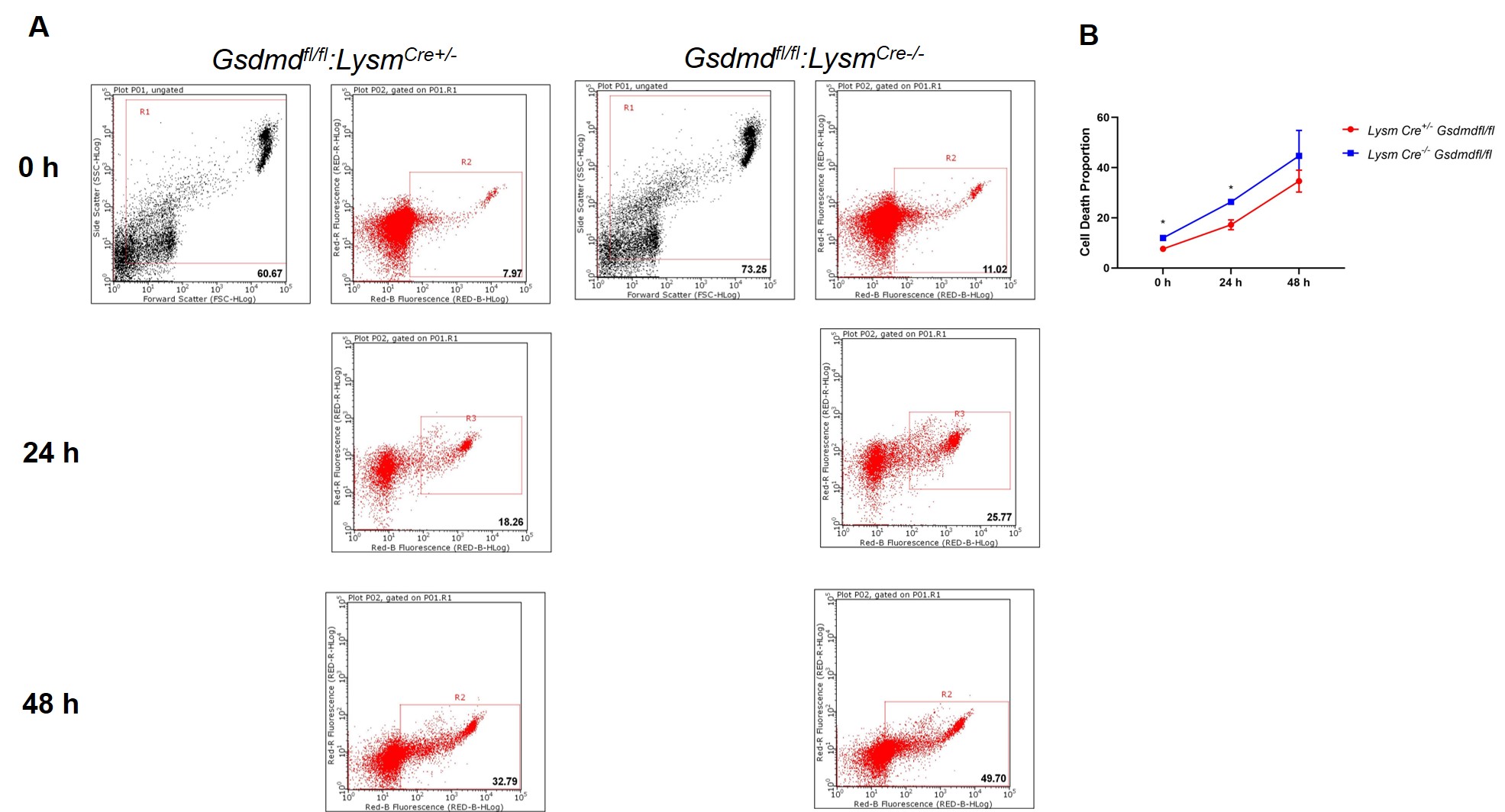


Supplemental Figure S11. Isolation of neutrophils from *Gsdmd^fl/fl^:Lysm^Cre+/-^* mice and littermate control mice at different time points of culture, represented by PI staining flow cytometry analysis results (A), and statistical results (B).
